# Complete Assembly of the Genome of an *Acidovorax citrulli* Strain Reveals a Naturally Occurring Plasmid in This Species

**DOI:** 10.3389/fmicb.2019.01400

**Published:** 2019-06-20

**Authors:** Rongzhi Yang, Diego Santos Garcia, Francisco Pérez Montaño, Gustavo Mateus da Silva, Mei Zhao, Irene Jiménez Guerrero, Tally Rosenberg, Gong Chen, Inbar Plaschkes, Shai Morin, Ron Walcott, Saul Burdman

**Affiliations:** ^1^Department of Plant Pathology and Microbiology, The Robert H. Smith Faculty of Agriculture, Food and Environment, The Hebrew University of Jerusalem, Rehovot, Israel; ^2^Department of Entomology, The Robert H. Smith Faculty of Agriculture, Food and Environment, The Hebrew University of Jerusalem, Rehovot, Israel; ^3^Department of Microbiology, University of Seville, Seville, Spain; ^4^Department of Plant Pathology, University of Georgia, Athens, GA, United States; ^5^Bioinformatics Unit, The Robert H. Smith Faculty of Agriculture, Food and Environment, The Hebrew University of Jerusalem, Rehovot, Israel

**Keywords:** *Acidovorax citrulli*, bacterial fruit blotch, SMRT sequencing, plasmid, toxin-antitoxin system

## Abstract

*Acidovorax citrulli* is the causal agent of bacterial fruit blotch (BFB), a serious threat to cucurbit crop production worldwide. Based on genetic and phenotypic properties, *A. citrulli* strains are divided into two major groups: group I strains have been generally isolated from melon and other non-watermelon cucurbits, while group II strains are closely associated with watermelon. In a previous study, we reported the genome of the group I model strain, M6. At that time, the M6 genome was sequenced by MiSeq Illumina technology, with reads assembled into 139 contigs. Here, we report the assembly of the M6 genome following sequencing with PacBio technology. This approach not only allowed full assembly of the M6 genome, but it also revealed the occurrence of a ∼53 kb plasmid. The M6 plasmid, named pACM6, was further confirmed by plasmid extraction, Southern-blot analysis of restricted fragments and obtention of M6-derivative cured strains. pACM6 occurs at low copy numbers (average of ∼4.1 ± 1.3 chromosome equivalents) in *A. citrulli* M6 and contains 63 open reading frames (ORFs), most of which (55.6%) encoding hypothetical proteins. The plasmid contains several genes encoding type IV secretion components, and typical plasmid-borne genes involved in plasmid maintenance, replication and transfer. The plasmid also carries an operon encoding homologs of a Fic-VbhA toxin-antitoxin (TA) module. Transcriptome data from *A. citrulli* M6 revealed that, under the tested conditions, the genes encoding the components of this TA system are among the highest expressed genes in pACM6. Whether this TA module plays a role in pACM6 maintenance is still to be determined. Leaf infiltration and seed transmission assays revealed that, under tested conditions, the loss of pACM6 did not affect the virulence of *A. citrulli* M6. We also show that pACM6 or similar plasmids are present in several group I strains, but absent in all tested group II strains of *A. citrulli*.

## Background

The genus *Acidovorax*, belonging to the Betaproteobacteria class, includes a variety of species that exhibit distinctive lifestyles. While some *Acidovorax* species are well adapted to water and soil environments, others have developed intimate relationships with eukaryotic organisms, including plants (Rosenberg et al., 2015). Among the latter, seven species were found to cause diseases in diverse plant families: *Acidovorax citrulli*, *Acidovorax avenae*, *Acidovorax oryzae*, *Acidovorax cattleyae*, *Acidovorax konjaci*, *Acidovorax anthurii*, and *Acidovorax valerianellae* (Willems et al., 1992; Gardan et al., 2000, 2003; Schaad et al., 2008). Of these, *A. citrulli*, causing bacterial fruit blotch (BFB) of cucurbits and *A. avenae*/*A. oryzae* causing several diseases on gramineous species, have been studied in the greatest detail (Rosenberg et al., 2015).

Bacterial fruit blotch garnered significant attention about 30 years ago after devastating outbreaks occurred in watermelon fields in the United States (Latin and Rane, 1990; Somodi et al., 1991; Schaad et al., 2003). Shortly thereafter, BFB was reported in many countries around the world. It is likely that this widespread distribution was facilitated by international seed trade, leading to global BFB outbreaks in cucurbit crops, including watermelon, melon, squash, pumpkin, and cucumber (Bahar and Burdman, 2010; Burdman and Walcott, 2012). Today, *A. citrulli* is a serious threat to global cucurbit production, especially melon and watermelon. The unavailability of effective tools for managing BFB, including the lack of sources of disease resistance, and the disease’s high destructive potential exacerbate the threat BFB poses to the seed industry, commercial fruit producers, and governmental regulatory agencies (Burdman and Walcott, 2012; Zhao and Walcott, 2018).

Based on genetic and biochemical characteristics, most *A. citrulli* strains can be divided into two well-differentiated groups. Group I comprises strains that have been mainly isolated from melon and other non-watermelon cucurbits, and are moderately to highly aggressive to a wide range of cucurbit crop species. In contrast, group II strains have been mainly isolated from watermelon, are highly virulent to this plant, but are less aggressive on other cucurbits (Walcott et al., 2000, 2004; Burdman et al., 2005; Feng et al., 2009; Silva et al., 2016; Zivanovic and Walcott, 2017). *A. citrulli* pathogenicity relies on a functional type III-secretion system (Bahar and Burdman, 2010; Johnson et al., 2011). We have shown that group I and II strains of *A. citrulli* are distinguishable based on the arsenal and sequences of type III-secreted virulence effectors they possess (Eckshtain-Levi et al., 2014).

In Eckshtain-Levi et al. (2016), we reported the genome sequence of a group I strain of *A. citrulli*, M6 (NCBI BioProject PRJNA298286). This strain was isolated in 2002 from a BFB outbreak on melons in Israel (Burdman et al., 2005) and subsequently became a model group I strain for fundamental and applied investigations of BFB. The M6 genome was sequenced using MiSeq Illumina technology, yielding a draft genome comprised of 139 contigs with an approximate size of 4.82 Mb. The M6 genome was ∼500 kb shorter than that of the group II model strain, AAC00-1 (NCBI accession NC_008752.1), sequenced by the Joint Genome Institute. Comparative analysis of the two strains indicated that this difference was mainly explained by eight ∼35–120 Kb fragments distributed throughout the AAC00-1 genome, which are absent in M6. Further PCR and BLAST analyses with other *A. citrulli* strains supported that some portions of these fragments differentiate group I and II strains of *A. citrulli* (Eckshtain-Levi et al., 2016). In addition to the M6 and AAC00-1 genomes, five additional genomes of *A. citrulli* strains are available in the public database. These are the draft genomes of the group I strains pslb65 (NCBI BioProject PRJNA274889; Wang et al., 2015a), ZJU1106 (NCBI BioProject PRJNA175738), DSM 17060 (BioProject PRJEB15996), and tw6 (BioProject PRJNA270710; Wang et al., 2015b), and the complete genome of the group II strain KACC17005 (NCBI accession NZ_CP023687.1; Park et al., 2017).

Here, we report the full assembly of the *A. citrulli* M6 genome after sequencing with Pacific Biosciences (PacBio) technology. This approach allowed us not only to close the M6 genome, but to identify a plasmid, pACM6, that was not detected in the assembly of the MiSeq reads. We also show that this or similar plasmids are present in other group I strains but absent in all tested group II strains.

## Results and Discussion

### Full Assembly of the *A. citrulli* M6 Genome Following PacBio Sequencing

A MiSeq-generated sequence of the *A. citrulli* group I strain M6, yielded a ∼4.82 MB draft genome distributed in 139 contigs (Eckshtain-Levi et al., 2016). Since M6 is the most studied *A. citrulli* group I strain, there is interest in the complete assembly of its genome. The emergence of third generation sequencing (TGS) platforms like PacBio allowed us to achieve this objective. PacBio sequencing, also referred to as single-molecule real-time (SMRT) sequencing, provides longer read lengths than second generation sequencing (SGS) technologies like MiSeq. This allows the closure of gaps between contigs of complex genomes (Bachall, 2009). In this study, we sequenced the *A. citrulli* M6 genome by PacBio, and used the existing MiSeq sequence data to make corrections, as needed. The power of utilization of hybrid-sequencing strategies, characterized by complementary combination of TGS and SGS technologies to overcome the limitation of each individual approach is well recognized (Rhoads and Au, 2015).

**FIGURE 1:**
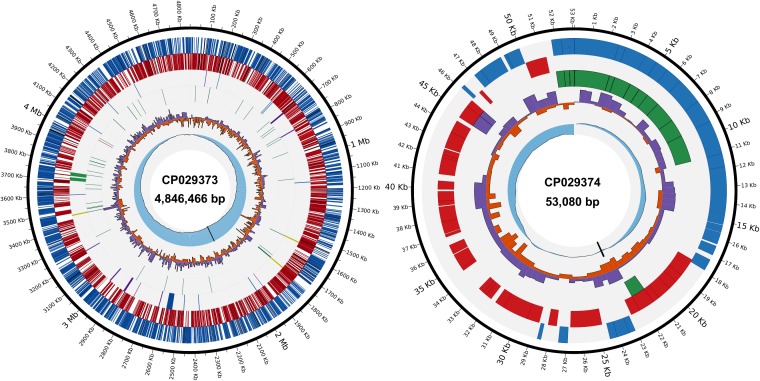
Circular views of the *Acidovorax citrulli* M6 chromosome and pACM6 plasmid. Chromosome (left), inner to outer tracks: (i), cumulative GC skew (blue) and replication origin (black); (ii), positive (purple) and negative (orange) GC skew; (iii), transfer (green) and transfer-messenger (red) RNA genes; (iv), ribosomal RNA (yellow) genes, type III secretion system (*hrp* cluster) and putative type III-secreted effector genes (blue), type IV pilus genes (purple) and prophages (green); (v), complementary strand genes (red); and (vi), direct strand genes (blue). In (v) and (vi), pseudogenes are shown in black. Plasmid (right), inner to outer tracks: (i), cumulative GC skew and replication origin; (ii), positive and negative GC skew; (iii), *vbhT*/*ficA* toxin-antitoxin system (purple), and type IV secretion system genes (green); (iv), complementary strand genes; and (v), direct strand genes. CP029373 and CP029374 are the accession numbers of the chromosome and the plasmid, respectively, as assigned by GenBank.

Assembly of the *A. citrulli* M6 PacBio reads resulted in two circular molecules with lengths of 4,846,466 bp and 53,080 bp (Figure 1), strongly supporting that this bacterium possesses a ∼53 kb plasmid. The average coverage of the two molecules was 232X for the chromosome and 173X for the putative plasmid. While the reduced coverage of the putative plasmid relative to the chromosome, could be due to some level of plasmid loss during bacterial growth (plasmid curing) or DNA extraction, it also indicated that the plasmid occurs at low copy number, which was further confirmed (see below). The relatively high coverage of the PacBio reads yielded a high-quality sequence. Comparison of the consensus sequence generated by PacBio and MiSeq reads did not reveal miss-called bases in the chromosome sequence, and only two miss-callings were detected and corrected in the sequence of the predicted plasmid. The genome annotation yielded a total of 4,216 open reading frames (ORFs): 4,153 in the chromosome and 63 in the predicted plasmid.

The general features of the *A. citrulli* M6 chromosome and plasmid sequences are shown in Table 1. The chromosomal locations of some genes of interest, including genes encoding type III secretion components and associated effectors, and type IV pilus genes, are shown in Figure 1 (for the plasmid, see below). Upon submission of this report, the updated genome and raw reads were submitted to NCBI under BioProject PRJNA298286. The closed chromosome and plasmid have been deposited under NCBI accession numbers CP029373 and CP029374, respectively, and the PacBio reads have been deposited in the NCBI Sequence Read Archive (SRA) archive under the accession number SRR7524637.

**Table 1:** Features of the assembled genome of *Acidovorax citrulli* M6 following sequencing with PacBio, assembly with Canu, and annotation with Prokka.

Feature	Chromosome	Plasmid
Size (bp)	4,846,466	53,080
No. of circular contigs	1	1
Genome G + C content (%)	68.9%	61.2%
No. of ORFs	4153	63
No. (and %) of hypothetical proteins	1630 (39.25%)	35 (55.6%)
Average ORF size (bp)	1027	739.1
Average ORF G+C content (%)	69.4	61.9
Coding percentage	88	87.7
No. of pseudogenes	243	0
No. of RNA genes (16S/5S/23S)	3/3/3	0
No. of tRNA genes	53	0
Other RNA	1	0
Prophage regions (regions/total bp)	2/41,148	0
Average effective number of codons (Nc)	33.05 ± 5.54	47.08 ± 6.21
Average codon adaptation index (CAI)	0.59 ± 0.14	0.25 ± 0.08
Average tRNA adaptation index (tAI)	0.43 ± 0.05	0.3 ± 0.06

*± standard deviation.*

### Annotation Evidence for a Plasmid in *A. citrulli* M6

Annotation of the putative plasmid revealed a higher percentage of genes encoding hypothetical proteins, relative to the chromosome (Table 1). Plasmids may possess large numbers of accessory genes with unknown or hypothetical functions, making their annotation difficult (Smalla et al., 2015). Additional features that characterize plasmids and other mobile genetic elements are differences in guanine-cytosine (GC) content and codon usage bias, relative to the bacterial chromosome (Tuller, 2011; Nishida, 2012; Shintani et al., 2015). In this regard, the predicted M6 plasmid has a lower GC content than the chromosome (61.2 and 68.9%, respectively). Also, the plasmid has a lower codon usage bias than the chromosome [measured as the effective number of codons (Nc; Table 1)], and does not have the completely adapted pool of transfer RNAs present in *A. citrulli* M6 [codon adaptation Index (CAI) and tRNA adaptation index (tAI); Table 1]. These parameters reflect the adaptation of the different genes to the available pool of encoded tRNAs and they have been used to assess the adaptation of acquired plasmids to their host (Blattner et al., 1997; Castillo-Ramírez et al., 2009). In general, constitutively and/or highly expressed genes are highly adapted to the tRNA pool. Recently horizontally acquired genes can reflect their previous host tRNA pool, hence their Nc/CAI/tAI will be different from genes with a longer evolutionary history in the new host (Blattner et al., 1997; Castillo-Ramírez et al., 2009).

The full annotation of the predicted plasmid is shown in Table 2, and the locations of some of the genes are highlighted in Figure 1. The annotation revealed genes that are strongly associated with plasmid replication, maintenance and transfer, such as *stbB* (plasmid stability protein), *repA* (plasmid replication protein), *parA* and *parB* (partitioning proteins), *traX* (conjugal transfer protein), and *mobC* (plasmid mobilization relaxosome protein) genes (Baxter and Funnell, 2014; Harmer and Hall, 2015; Shintani et al., 2015). Another gene annotated in the predicted plasmid is *ssb* that encodes a protein belonging to the primosome PriB/single-strand DNA-binding family (Interpro IPR000424). *ssb* genes are often localized in a variety of self-transmissible plasmids (Meyer and Laine, 1990). Ssb proteins can bind to single-stranded DNA and play important roles in DNA replication, recombination and repair (Zavitz et al., 1991).

**Table 2:** Genes annotated in the *Acidovorax citrulli* M6 plasmid pACM6 by Prokka.

Locus tag	Gene name	Product/Function^a^
APS58_p00001	*virB4*	T4SS protein virB4
APS58_p00002		hypothetical protein
APS58_p00003	*virB6*	T4SS protein TrbL/VirB6
APS58_p00004	*virB5*	T4SS protein VirB5
APS58_p00005	*virB8*	T4SS protein VirB8
APS58_p00006	*virB9*	T4SS protein VirB9
APS58_p00007	*virB10*	T4SS protein VirB10
APS58_p00008	*virB11*	T4SS protein VirB11
APS58_p00009	*traG*	conjugal transfer protein TraG
APS58_p00010	*ompA*	T4SS putative outer membrane lipoprotein
APS58_p00011		hypothetical protein
APS58_p00012		hypothetical protein
APS58_p00013	*ssb*	single-stranded DNA-binding protein Ssb
APS58_p00014		hypothetical protein
APS58_p00015		hypothetical protein
APS58_p00016		hypothetical protein
APS58_p00017		hypothetical protein
APS58_p00018	*yncB*	endonuclease YncB
APS58_p00019		hypothetical protein
APS58_p00020		hypothetical protein
APS58_p00021		hypothetical protein
APS58_p00022	*virD2*	T4SS T-DNA border endonuclease VirD2
APS58_p00023	*mobC*	plasmid mobilization relaxosome protein MobC
APS58_p00024		hypothetical protein
APS58_p00025	*stbB*	plasmid stability protein StbB
APS58_p00026		hypothetical protein
APS58_p00027	*parB1*	putative chromosome partitioning protein ParB
APS58_p00028		hypothetical protein
APS58_p00029		hypothetical protein
APS58_p00030		hypothetical protein
APS58_p00031		hypothetical protein
APS58_p00032		hypothetical protein
APS58_p00033		hypothetical protein
APS58_p00034		hypothetical protein
APS58_p00035		hypothetical protein
APS58_p00036		hypothetical protein
APS58_p00037		hypothetical protein
APS58_p00038		hypothetical protein
APS58_p00039		hypothetical protein
APS58_p00040		hypothetical protein
APS58_p00041		hypothetical protein
APS58_p00042	*traX*	conjugal transfer protein TraX
APS58_p00043		KfrA_N and SMC_N domain-containing protein
APS58_p00044		hypothetical protein
APS58_p00045		hypothetical protein
APS58_p00046		hypothetical protein
APS58_p00047	*repA*	plasmid replication protein RepA
APS58_p00048		hypothetical protein
APS58_p00049		sigma-70 family RNA polymerase sigma factor
APS58_p00050	*parB2*	putative chromosome partitioning protein ParB
APS58_p00051	*parA*	chromosome partitioning protein ParA
APS58_p00052	*fic*	adenosine monophosphate-protein transferase Fic, toxin VbhT
APS58_p00053	*vbhA*	antitoxin protein VbhA
APS58_p00054		hypothetical protein
APS58_p00055		hypothetical protein
APS58_p00056	*umuD*	translesion error-prone DNA polymerase V autoproteolytic subunit
APS58_p00057	*dinB*	DNA polymerase IV
APS58_p00058		hypothetical protein
APS58_p00059		hypothetical protein
APS58_p00060		hypothetical protein
APS58_p00061	*virB1*	T4SS protein VirB1
APS58_p00062	*virB2*	T4SS protein VirB2
APS58_p00063	*virB3*	T4SS protein VirB3


*^a^T4SS, type IV secretion system*.

Additional genes that are frequently located in plasmids and were detected in the predicted plasmid are the *vir* genes, which encode components of a type IV/conjugal transfer secretion system. Type IV secretion systems are large protein complexes, often encoded on self-transmissible plasmids that span the cell envelope of many bacteria and mediate translocation of proteins and protein-DNA complexes (Wallden et al., 2010). Based on the M6 plasmid annotation (Table 2), it seems that this plasmid contains all genes required for the synthesis of a functional type IV secretion system.

The *A. citrulli* M6 plasmid contains an operon encoding components of a putative toxin-antitoxin (TA) module: *APS58_p00052*, encoding the toxin, adenosine monophosphate-protein transferase Fic (for filamentation induced by cAMP), and *APS58_p00053*, encoding the antitoxin protein VbhA (Table 2). TA systems were first identified in the mid-1980s as post-segregational killing mechanisms involved in plasmid maintenance (Gerdes et al., 1986). Later, additional functions were attributed to TA systems including roles in stress management, gene regulation and virulence (Goeders and Van Melderen, 2014). The toxin protein encoded in the M6 plasmid belongs to the Fic/Doc family (PFAM02661). Toxins in this family are often named Fic, VbhT and Doc, and their cognate antitoxins are named FicA, VbhA and Phd. The Fic-VbhA TA system has been well-studied in *Bartonella schoenbuchensis*. It was proposed that in this bacterium Fic exerts toxicity through adenylylation of target proteins, while the antitoxin VbhA inhibits Fic toxicity by forming a tight complex with the latter (Engel et al., 2012; Goepfert et al., 2013). This class of TA modules is widespread in gram-positive and gram-negative bacteria. Recently, Shidore and Triplett (2017) reported that genes encoding Fic/Doc TA systems are present in several strains of plant-associated bacteria, including *Clavibacter michiganensis* subsp. *michiganensis*, *Agrobacterium tumefaciens*, *Xylella fastidiosa*, *Xanthomonas oryzae* and *Xanthomonas citri*, and the legume symbionts *Mesorhizobium loti*, and *Rhizobium leguminosarum*. Previously, Goepfert et al. (2013) reported the presence of a Fic-VbhA module in the plant growth-promoting bacterium, *Pseudomonas fluorescens*.

A different TA system, belonging to the VapB-VapC family, was recently reported in *A. citrulli* (Shavit et al., 2016). This TA system was present in all assessed group II strains of *A. citrulli*, but absent in all tested group I strains, including M6. As we show below, based on plasmid overlap analyses of *A. citrulli* strains, the Fic-VbhA TA system encoded in the M6 plasmid is characteristic of some group I, but no group II strains.

Interestingly, the M6 plasmid also contains an operon comprising genes *APS58_p00056* and *APS58_p00057*, which encode UmuD (translesion error-prone DNA polymerase V autoproteolytic subunit) and DinB (DNA polymerase IV), respectively (Table 2). These are important components of the SOS system, which is induced under DNA-damaging conditions and is characterized by expression of genes involved in DNA-damage tolerance, DNA repair and mutagenesis (Little and Mount, 1982; Maslowska et al., 2018). The SOS response is repressed by LexA, which under regular conditions binds to the so-called SOS boxes in the promoter region of SOS response genes interfering with RNA polymerase (Little et al., 1981). Under DNA damage-inducing conditions, RecA inactivates the LexA repressor inducing expression of SOS regulon genes (Little, 1993). The *A. citrulli* M6 chromosome has a *lexA* gene (*APS58_0003*), which possesses high levels of identity with *lexA* genes from other *Acidovorax* species and members of the Betaproteobacteria. In most Betaproteobacteria, the SOS box consensus sequence is CTGT-N8-ACAG (Sanchez-Alberola et al., 2015). We used this motif to screen for putative SOS boxes in the M6 genome using fuzznuc. While we found several SOS-boxes in the chromosome in promoter regions of typical SOS-related genes (e.g., recombinases, DNA modification/repair proteins, ATP-dependent DNA helicase and radical SAM proteins among others; not shown), only one SOS box was detected in the plasmid. This box is located in positions 47052–47067, spanning the near upstream region of the *umuD-dinB* operon and the beginning of the *umuD* coding sequence. In the future it will be interesting to investigate the SOS response in *A. citrulli*, including the involvement of the plasmid genes *umuD* and *dinB*. Interestingly, UmuD/DinB possess similar functions to RulA/RulB that in *Escherichia coli* are important for ultraviolet (UV) tolerance (Smith and Walker, 1998). Moreover, plasmid-encoded *rulAB* operons are widely distributed in several *Pseudomonas syringae* pathovars, in which they were shown to contribute to UV tolerance and epiphytic fitness (Sundin and Murillo, 1999; Sundin et al., 2000). Since *A. citrulli* is a foliar pathogen that is commonly exposed to UV radiation, it will be interesting to assess whether pACM6, and particularly, the *umuD-dinB* operon contributes to epiphytic fitness of this bacterium. It will also be worth assessing whether in *A. citrulli* the SOS response is connected with regulation of genes encoding type III-secreted effectors, as shown for other plant-pathogenic bacteria (Jackson et al., 2011).

### Confirmation of Plasmid pACM6 in *A. citrulli* M6

To verify the presence of the predicted plasmid in *A. citrulli* M6, we sought to visualize it by gel electrophoresis. We tried several procedures including a commercial kit (Qiagen Plasmid Maxi kit) and those described by Espuny et al. (1987) and Birnboim and Doly (1979). Negative controls included *E. coli* DH5a and the *A. citrulli* group II strain, AAC00-1, which based on its complete assembly, is not predicted to carry a plasmid. Among the tested procedures, the Birnboim and Doly (1979) method was the most efficient, revealing a faint band indicative of a plasmid in strain M6 in most extractions (Supplementary Figure S1). As expected, no plasmid bands were detected for *A. citrulli* AAC00-1 and *E. coli* DH5a. With that said, detection of a plasmid-like band in extracts from strain M6 could not be achieved in all repetitions. These inconsistencies could be due to technical limitations and/or low plasmid copy number.

**FIGURE 2:**
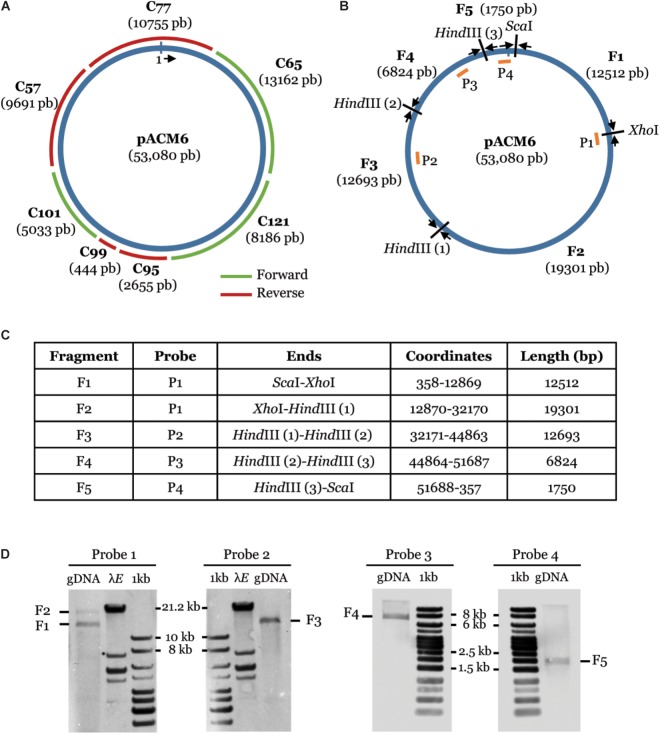
Confirmation of plasmid pACM6 in *A. citrulli* M6. **(A)** Partial overlap (94%) of the pACM6 sequence obtained from the PacBio sequencing (inner circle) with seven full contigs generated from the MiSeq sequencing (outer fragments; Eckshtain-Levi et al., 2016). **(B–D)** Confirmation of the circular nature of pACM6 by Southern blot analysis and PCR. **(B)** Circular map of pACM6 showing the location of *Sca*I, *Xho*I, and *Hind*III restriction sites, the resulting fragments (F1 to F5) after restriction with these enzymes, selected probes for Southern blot analysis (P1 to P4, shown as orange fragments within the circular map), and PCR primers for amplification of the junctions between restricted fragments (black arrows). PCR primers are listed in Supplementary Table S1 and PCR results are shown in Supplementary Figure S2. **(C)** Coordinates and sizes of the restricted fragments. **(D)** Detection of fragments F1 to F5 by Southern blot with probes P1 to P4. gDNA, genomic DNA of *A. citrulli* M6 treated with *Sca*I, *Xho*I, and *Hind*III; aaa*E*, aaa*Eco*RI marker (Thermo Fisher Scientific); 1 kb, Generuler 1-kb DNA Ladder (Fermentas).

To further verify plasmid occurrence in strain M6, we first mapped the 139 contigs of the M6 draft genome assembled from the MiSeq sequencing (Eckshtain-Levi et al., 2016) on the plasmid sequence. The plasmid was covered by seven contigs, ranging from 444 to 13,162 bp, with a total overlap of 94% (Figure 2A). Importantly, these seven contigs fully mapped on the plasmid (namely, they did not include chromosomal regions). As an additional approach to verify the circular nature of the plasmid, we combined Southern blot hybridization and PCR. Total DNA of strain M6 was digested with restriction enzymes *Sca*I, *Xho*I, and *Hind*III. If the predicted plasmid indeed occurs, this treatment was expected to yield five plasmid-derived fragments with the following approximate sizes: 1.7, 6.8, 12.5, 12.7, and 19.3 kb (Figure 2B,C). Four probes, ranging from 1,039 to 1,147 bp, and targeting the aforementioned fragments were generated by PCR using primers described in Supplementary Table S1. Southern blot analysis using these probes on M6 *Sca*I/*Xho*I/*Hind*III-digested DNA yielded fragments with the expected sizes (Figure 2D). To fully demonstrate the circular nature of this element, PCR primers were designed to amplify 491 to 631 bp from regions around the junctions between the fragments (Supplementary Table S1). All PCR combinations yielded products with the expected sizes (Supplementary Figure S2). The resulting PCR fragments were verified by sequencing and matched, at 100% identity, with the target regions in the plasmid sequence. Overall, these results confirmed that *A. citrulli* M6 has a ∼53 kb plasmid, which we named pACM6.

With regards to plant-pathogenic *Acidovorax* species, a ∼59.5 kb plasmid, named pAAA83, was reported in the *A. avenae* strain, 83 (Yoshii et al., 2015). Comparative analysis of pACM6 and pAAA83 did not yield any significant alignment. Based on NCBI data, plasmids have been also detected in environmental strains of *Acidovorax* sp., like the nitroaromatic compound-degrading strain JS42 and the polycyclic aromatic hydrocarbon (PAH)-degrading strains NA2, P3, and P4. One ∼3 kb plasmid carrying four ORFs was reported in an environmental isolate of *Acidovorax temperans*, CB2 (Boycheva et al., 2015).

### Plasmids Similar to pACM6 Occur in Several Group I Strains of *A. citrulli*

We screened other group I and II *A. citrulli* strains for pACM6-like plasmids. BlastN was used to determine the percent overlap of pACM6 in 19 *A. citrulli* genomes from a proprietary collection of draft sequences, to which we have access. This collection is composed of draft genomes of eleven group I and eight group II strains that were sequenced by a private company (anonymity requested) and belong to various haplotypes based on DNA fingerprinting generated by pulsed field gel electrophoresis (PFGE) of *Spe*I-digested DNA (Walcott et al., 2004; Zhao and Walcott, 2018). The overlap of pACM6 in seven group I strains, belonging to three different haplotypes (B5, B9, and B12), ranged from 83.8 to 96.0% (Table 3). Images of overlap distribution of some of these strains are shown in Supplementary Figure S3. It is worth noting that the overlap between pACM6 and the corresponding contigs of M6 assembled from MiSeq was 94%. Therefore, these results strongly support the occurrence of pACM6 or pACM6-like plasmids in these strains. Moreover, as similar as observed for strain M6 (Figure 2A), the draft contigs of the group I strains that overlapped with pACM6, fully matched with pACM6 regions, further strengthening plasmid occurrence in these strains. Also important is the fact that the percentages of identity obtained for the alignments between pACM6 and the corresponding contigs of the draft genomes of the aforementioned strains were high, ranging between 97.4 and 99.9% (and 99.9% in most cases; Table 3). Therefore, while we use the term “pACM6-like plasmids” because we analyzed draft, incomplete genomes, there is strong evidence that the plasmids in these strains are identical or almost identical to pACM6. In contrast, no overlap was observed between pACM6 and contigs from four group I *A. citrulli* strains that belonged to PFGE haplotypes B3, B8 and B13, and from the eight group II strains that belonged to seven PFGE haplotypes: A2, A3, A4, A5, A11, A26, and A28 (Table 3).

**Table 3:** *Acidovorax citrulli* strains tested by PCR with sets of primers targeting pACM6 primer sets (pACM6-F1/pACM6-R1 and pACM6-F2/pACM6-R2) and/or tested for overlap with pACM6 by BlastN^a^.

Group	PFGE haplotype^b^	Strain^c^	Country	PCR results	Overlap and identity (%)^d^
I	B1	92-301	United States	Negative	–
	B2	92-305	United States	Negative	–
	B3	92-300	United States	Negative	–
		213-50	United States	Negative	0
	B4	AU-2	Australia	Negative	–
	B5	209-108	China	Positive	–
		213-58	Chile	Positive	92.8 (99.9)
		213-59	China	Positive	92.8 (99.9)
		213-60	Unknown	Positive	96.0 (97.8)
		213-61	Mexico	Positive	92.1 (99.9)
	B6	98-17	United States	Negative	–
	B7	99-5		Negative	–
	B8	1ACANT58-1	United States	Negative	–
		213-51	China	Negative	0
	B9	213-55	Unknown	Positive	92.5 (99.9)
		213-56	China	Positive	89.1 (99.9)
		213-57	Thailand	Positive	–
	B10	200-30	United States	Positive	–
	B11	201-14	Australia	Negative	–
	B12	213-54	Unknown	Positive	83.8 (97.4)
	B13	203-16	Honduras	Positive	–
		213-52	Unknown	Negative	0
		213-53	Brazil	Negative	0
	B14	208-1	India	Negative	–
	B15	207-41	Unknown	Negative	–
	B16	206-2	Unknown	Negative	–
	B17	206-1	Unknown	Negative	–
	B20	208-26	Georgia	Negative	–
	B21	M6	Israel	Positive	100.0
II	A1	AAC00-1	United States	Negative	0
		92-2	United States	Negative	–
	A2	92-303	United States	Negative	–
		213-41	Unknown	Negative	0
		213-47	Thailand	Negative	0
	A3	213-44	Unknown	Negative	0
	A4	92-3	United States	Negative	–
		213-42	Unknown	Negative	0
	A5	94-55	United States	Negative	–
		213-48	Unknown	Negative	0
	A6	96-6	United States	Negative	–
	A7	94-39	United States	Negative	–
	A11	94-12	United States	Negative	–
		213-46	Unknown	Negative	0
	A12	203-65	Unknown	Negative	–
	A13	206-102	China	Negative	–
	A14	206-103	China	Negative	–
		211-8	United States	Negative	–
	A16	205-14	United States	Negative	–
	A17	211-36	United States	Negative	–
	A22	206-79	China	Negative	–
	A24	211-2	United States	Negative	–
		211-29	Unknown	Negative	–
	A26	213-49	Unknown	Negative	0
	A28	213-45	Unknown	Negative	0
	A29	208-10	Hungary	Negative	–
	A30	209-118	China	Negative	–


*^a^Overlap between pACM6 and genome sequences of A. citrulli strains was tested on a proprietary collection of A. citrulli draft genomes, except for strains AAC00-1 (NCBI accession NC_008752.1) and M6 (positive control). ^b^Haplotypes based on PFGE analysis following digestion with SpeI (Eckshtain-Levi et al., 2016; Zhao and Walcott, 2018). ^c^Strain M6 is from the S. Burdman collection. The other strains are from the R. Walcott collection. ^d^– means not-determined. In cases showing significant percentages of overlap, the pairwise % identity is indicated between parentheses*.

In agreement with the above findings, among the *A. citrulli* genomes available in the public database, only two genomes, of the group I strains pslb65 and ZJU1106, had contigs that mapped to the pACM6 sequence. In the case of pslb65, one single contig fully mapped to pACM6 at 100% overlap and 99% identity. Regarding ZJU1106, pACM6 was fully covered by five entire contigs at identity levels of 99–100%. These results support the occurrence of pACM6 or like plasmids in these strains. In contrast, no significant hits matching with pACM6 were found in the group II strains AAC00-1 and KACC17005, and in the group I strains tw6 and DSM 17060. We have previously reported that, among the group I genomes that were available in 2016, strain M6 was more closely related to strain pslb65 than to strain tw6 (Eckshtain-Levi et al., 2016). The closest relatedness between strain M6 and pslb65 among all available group I genomes is also well-illustrated by an NCBI dendrogram based on Blast of publically available *A. citrulli* genomes^1^.

We also tested the aforementioned strains and others representing additional PFGE haplotypes by PCR assays using two primer sets targeting the plasmid, pACM6-F1/pACM6-R1 and pACM6-F2/pACM6-R2 (Supplementary Table S1). Fifty-five strains were tested, 28 from group I and 27 from group II. Overall, results from PCR reactions agreed with those from BlastN analyses (Table 3). Thirteen of 28 group I strains yielded positive reactions with both primer combinations. In agreement with BlastN results, five tested strains from haplotype B5, three strains from haplotype B9, and the tested strain from haplotype B12 yielded positive PCR reactions. These assays also yielded positive PCR reactions for strain 200-30, which belongs to PFGE haplotype B10 that was not represented in the BlastN analysis (Table 3).

Also in agreement with the BlastN results, two group I strains from haplotype B3 and two strains from haplotype B8 gave negative PCR results. On the other hand, while the two B13 strains (213-52 and 213-53) that showed no overlap with pACM6 gave negative PCR results, a different strain belonging to this haplotype (203-16) yielded positive PCR reactions with the two pACM6 primer sets (Table 3). Although we cannot discard a bias possibility (i.e., incomplete genome assembly of the first two strains), this result suggests that PFGE haplotype may not be fully indicative of presence/absence of a pACM6-like plasmid. PCR tests also yielded negative results for group I strains representing haplotypes B1, B2, B6, B11, B14, B15, B16, B17, and B20, that were not represented in the BlastN analyses (Table 3). Despite these results, we must be careful with our interpretations, since negative PCR results could occur if some or all primers did not sufficiently complement the template DNA.

No group II strains yielded positive PCR results with the pACM6 primer sets (Table 3). While BlastN analyses included strains representing 7 PFGE haplotypes from group II, PCR tests covered strains from 19 group II haplotypes. Overall, findings from BlastN analyses and PCR strongly support that pACM6 or pACM6-like plasmids occur in some group I strains, but not in group II strains.

### Correlation Between Genetic Relatedness and Occurrence of pACM6-Like Plasmids

We asked whether there is correlation between the genetic relatedness of group I strains and occurrence/absence of pACM6-like plasmids. First, we carried out multilocus sequence analysis (MLSA) including all available genomes (from NCBI and from the aforementioned proprietary collection) using seven housekeeping (HK) genes described by Feng et al. (2009) (*gmc*, *ugpB*, *pilT*, *lepA*, *trpB*, *gltA*, and *phaC*). In agreement with Feng et al. (2009) findings, almost no variability was found among the tested group I strains in these genes. Further, we assessed the variability among the sequenced strains of fifteen additional HK genes. Very low polymorphism was found also in these genes, thus impeding analysis of phylogenetic differentiation by this technique. As an alternative approach, we exploited the available knowledge on the diversity of *A. citrulli* haplotypes based on PFGE following macrorestriction of genomic DNA. This approach has been commonly used to evaluate the genetic diversity of *A. citrulli* isolates (Walcott et al., 2000, 2004; Burdman et al., 2005; Zhao and Walcott, 2018). Despite some limitation in terms of resolution, this method has been considered as a valid tool for evaluation of genetic relatedness among bacterial isolates/haplotypes (Struelens et al., 1992; Thorsteinsdottir et al., 2010; Allard et al., 2013).

**FIGURE 3:**
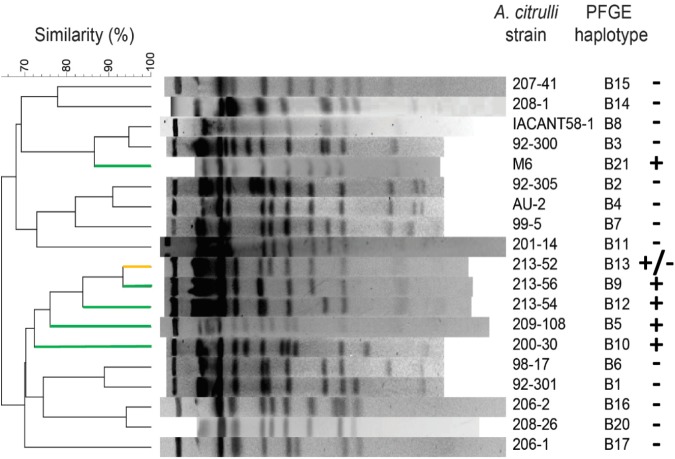
Occurrence of pACM6-like plasmids in group I haplotypes of *A. citrulli*. A dendrogram was generated following pulse-field gel electrophoresis of *Spe*I-digested genomic DNA of *A. citrulli* strains. The strains represent haplotypes assessed in this report for presence of pACM6-like plasmids by genome overlap analysis and/or PCR (see Table 3). Distance matrix was generated by Dice’s (1945) coefficient of similarity. The dendrogram was generated based on the unweighted pairwise group method with arithmetic mean (UPGMA) algorithm. + indicates occurrence of pACM6-like plasmid; – indicates absence of pACM6-like plasmid; +/– indicates that a pACM6-like plasmid was detected in one out of three strains tested in this haplotype. For better visualization, dendrogram lines corresponding to (+), and (+/–) haplotypes are shown in green and yellow, respectively.

A PFGE-based dendrogram showing the relatedness among the group I haplotypes that were assessed in this study for plasmid occurrence is shown in Figure 3. This dendrogram includes the vast majority of known group I haplotypes (Zhao and Walcott, 2018). The dendrogram shows that pACM6 or like plasmids are present in representative strains from two paraphyletic groups: one including haplotypes B5, B9, B10, B12 and B13, and the other including haplotype B21 to which strain M6 belongs. This picture suggests that pACM6 or like plasmids could have been acquired by different group I strains in at least two events of horizontal gene transfer. With that said, this assumption should be further verified by assessing more strains and by other approaches. As mentioned above, in the case of haplotype B13, one strain (203-16) was positive for the plasmid, while two (213-52 and 213-53) were negative (Table 3). The picture emerging from Figure 3, showing high genetic relatedness between this haplotype and haplotypes B5, B9, B10, and B12 that were positive for plasmid occurrence, suggests that pACM6 loss is possible. This notion was confirmed at continuation (see below).

### BLAST Analyses of pACM6 Against the Public Database

To gain some insight about the possible origin of pACM6 we carried out several Blast analyses of the plasmid sequence and ORFs against the public database at NCBI. Besides hits to *A. citrulli* proteins in some cases, BlastX against non-redundant protein sequences (nr) revealed some levels of similarity to proteins from bacteria from diverse phyla, but mainly from the Proteobacteria. In most cases, the levels of similarity varied from 40 to 65% for partial query overlaps. Among these, the most abundant hits belonged to the Betaproteobacteria class (e.g., other *Acidovorax* species and species belonging to genera *Azoarcus*, *Burkholderia*, *Bordetella*, *Ralstonia*, and *Comamonas* among others) followed by Gammaproteobacteria representatives including some *Xanthomonas* and *Pseudomonas* strains. However, the picture emerging from these analyses did not reveal a dominant genus with sequences closely related to pACM6.

We further determined the phylogenetic assignment of nine typical plasmid gene products by MEGAN analysis, after importing their multiple alignments from NCBI BlastP. This analysis was done after excluding the *A. citrulli* taxid from BlastP, since its inclusion masked any insight regarding phylogenetic relatedness. Results from MEGAN analysis were in agreement with Blast of pACM6 ORFs. The top species/genus hits for each of these proteins are shown in Supplementary Table S2. Of the nine proteins, five (Ssb, MobC, ParB_1, RepA and ParB_2) could be assigned to the Proteobacteria phylum, three (StbB, UmuD, and DinB) to the Betaproteobacteria class, and one (ParA) to the Burkholderiales order.

We also carried out BlastN analyses against whole-genome shotgun contigs (wgs) and the RefSeq Genome Database (refseq_genomes) targeting the Proteobacteria phylum. The only hits that showed high percentages of overlap and sequence identity were from *A. citrulli* contigs. As mentioned above, the pACM6 plasmid was almost fully covered by contigs of draft genomes of two group I *A. citrulli* strains, pslb65 and ZJU1106, in addition of M6 contigs generated from the MiSeq genome.

Overall, despite some relatedness to the Proteobacteria phylum and the Betaproteobacteria class, Blast and MEGAN analyses did not yield conclusive insights into the possible origin of pACM6. These analyses also indicate that, among sequenced genomes, this plasmid seems to be unique to *A. citrulli*.

### pACM6 Occurs at Low Copy Number and Can Be Eliminated From *A. citrulli* M6

The relatively low coverage of pACM6 following PacBio sequencing and assembly suggested that this plasmid occurs at low copy numbers. It also suggested that the plasmid could be lost in some cells within the population. To estimate the copy number of pACM6 we first carried out PCR reactions targeting the plasmid and the chromosome of serially diluted suspensions of *A. citrulli* M6. These assays showed a similar level of amplification of chromosome and plasmid targets, supporting the low copy number of pACM6 (not shown). Further, we estimated the plasmid copy number based on analysis of DNA extracts with three different chromosome primers pairs and three specific plasmid primer sets by quantitative real-time PCR (qRT-PCR), as described by Wang et al. (2016). This analysis indicated pACM6 has a ∼4.1 ± 1.3 chromosome equivalents. With that said, we should be careful with the interpretation of these results as some cells within the population could have lost the plasmid; therefore, we cannot exclude the possibility that some cells could carry more copies of pACM6. In addition, it is possible that the average copy number of pACM6 may vary under different conditions.

First attempts to test whether *A. citrulli* M6 can lose pACM6 were done following three cycles of growth of the bacterium in nutrient broth (NB) medium at 28°C (see section “Materials and Methods”). After the 3rd growth cycle, the cultures were serially diluted and plated on nutrient agar (NA) plates. Then, sample colonies were collected and tested by colony-PCR using plasmid and chromosomal primer sets. This procedure was repeated three time and a total of 450 colonies were tested, with no one yielding negative results for plasmid primers.

**FIGURE 4:**
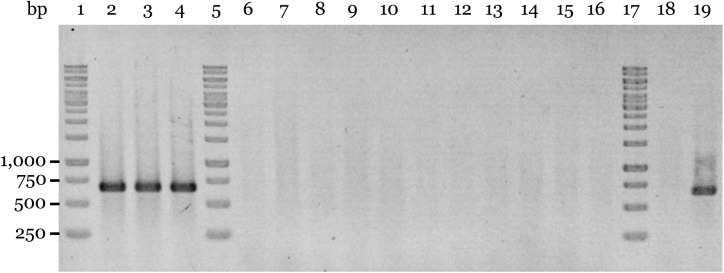
Assessment of plasmid curing of *A. citrulli* M6. Plasmid curing was achieved after incubation of *A. citrulli* M6 cultures at 41°C as described in the Section “Materials and Methods.” The colonies were tested by colony-PCR using primers pACM6-F2 and pACM6-R2 (Supplementary Table S1). Lines: 1, 5, and 17, Generuler 1-kb DNA Ladder (Fermentas); 2 to 4, non-cured isolates (collected after exposure to 41°C) M6-NC1 to M6-NC3, respectively; 6 to 16, pACM6-cured strains M6-PC1 to M6-PC11, respectively; 18, wild-type AAC00-1 (negative control); and line 19, wild-type M6.

It is well known that plasmid curing in bacteria may occur following exposure to elevated temperatures (Meletzus and Eichenlaub, 1991; Datta et al., 2006; Letchumanan et al., 2015). We therefore tried a similar procedure but incubating the bacteria at 41°C. Under these conditions, we were able to detect 11 out of 450 colonies (∼2.4%) that yielded negative colony-PCR results using the plasmid primer sets pACM6-F1/pACM6-R1 and pACM6-F2/pACM6-R2 (shown for the latter in Figure 4). These results were further confirmed by PCR tests using DNA extracts from these strains and other plasmid primer sets (shown for strain M6-PC1 in Supplementary Figure S2). Importantly, the pACM6-negative colonies yielded positive results in PCR reactions with the *A. citrulli* specific primers BX-L1/BX-S-R2 (Bahar et al., 2008; Supplementary Table S1) thus confirming their *A. citrulli* identity (not shown). Overall, these results confirmed that *A. citrulli* M6 cells can lose pACM6.

Although *A. citrulli* M6 can lose pACM6, it seems that this low-copy number plasmid is stably maintained in the bacterial population. As mentioned above, pACM6 carries a Fic-VbhA TA system. Among other functions, plasmid-located TA modules have been shown to be involved in plasmid maintenance (Gerdes et al., 1986). We recently conducted transcriptome profiling by RNA-Seq to characterize expression of *A. citrulli* M6 genes. Bacteria were grown for 72 h at 28°C in XVM2 medium that mimics to some extent the plant apoplast environment (Wengelnik et al., 1996). While the RNA-Seq data will be summarized in a future report, here we show results of pACM6 gene expression (Supplementary Figure S4). Interestingly, under tested conditions, *vbhA*, encoding the antitoxin, was the plasmid gene with the highest expression, and *fic*, encoding the concomitant toxin, was also among the highly expressed genes. While the Fic-VbhA TA module could play an important role in maintenance of pACM6 in the *A. citrulli* M6 population, this hypothesis should be further verified. It would be also interesting to measure the expression of the *fic-vbhA* genes under different conditions (eg., minimal and rich media at different growth stages and/or temperatures, *in planta* expression) and assess whether there is correlation between expression of these genes and the level of pACM6 loss.

### Loss of pACM6 Does Not Seem to Affect Growth and Virulence of *A. citrulli* M6

The obtention of pACM6-cured strains allowed us to assess whether this plasmid contributes to bacterial growth and virulence of *A. citrulli* M6. We compared three cured strains, M6-PC1, M6-PC5, and M6-PC8, with wild-type M6 for these abilities. These cured strains were isolated from three different rounds of plasmid curing experiments. In growth curve experiments and virulence assays, we also included three isolates, M6-NC1, M6-NC2, and M6-NC3, which were isolated after plasmid curing experiments (namely, after exposure to 41°C) but retained pACM6 (Figure 4). Results from growth curve experiments in rich (NB) and minimal (XVM2) media are shown in Supplementary Figure S5. In NB, the three tested cured strains consistently showed growth rates and maximal optical density values that were higher than wild-type M6. However, a similar growth pattern as for the cured strains was observed for the non-cured strain M6-NC3. In XVM2 medium, the three cured strains entered into the exponential phase faster than the non-cured strains and wild-type M6. However, the three non-cured strains also entered into the exponential phase faster than wild-type M6. In addition, in this medium, differences in growth patterns were observed among strains belonging to the same group (eg., cured and non-cured strains). Overall, these results indicate that the aggressive treatment used for curing (exposure to 41°C) appeared to have caused important changes in growth behavior. Therefore, while it is well established that fitness costs may be associated with plasmid carriage (Harrison et al., 2015; San Millan and MacLean, 2017; Carroll and Wong, 2018), further exploration is required to assess the effects of pACM6 on fitness of *A. citrulli*. This is a complex question since, as exemplified in the interaction between the large conjugative plasmid pQBR103 and *Pseudomonas* species, the dynamics of plasmid stability and fitness effects largely depends on the environment and on the genetic background of the bacterial host (Kottara et al., 2018).

**FIGURE 5:**
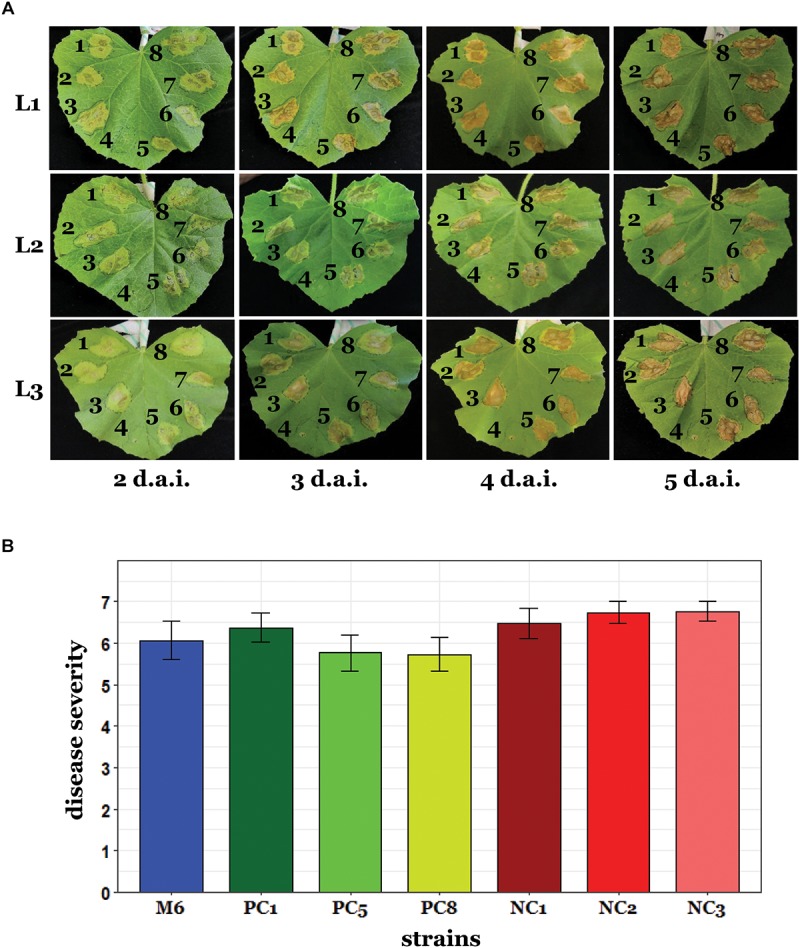
Virulence assays with *A. citrulli* M6 cured strains. **(A)** Leaf infiltration assays. Leaves of 3-week-old melon plants were infiltrated in their abaxial side with ∼10^6^ CFU/ml bacterial suspensions using a needleless syringe. The plants were kept in a greenhouse at 27–28°C and symptoms were recorded every day [since 2 days after inoculation (d.a.i.) when symptoms became visible] until 5 d.a.i. Pictures are shown for three representative leaves (L1 to L3), out of eight inoculated leaves. Tested strains were: M6 cured strains M6-PC1 (1), M6-PC5 (2), and M6-PC8 (3); an M6 mutant defected in the *hrcV* gene and impaired in pathogenicity (4); wild-type M6 (5); and three isolates, M6-NC1 (6), M6-NC2 (7), and M6-NC3 (8), which were collected from curing experiments after exposure to 41°C and were shown to retain pACM6. This experiment was carried out twice yielding similar results. **(B)** Seed transmission assays. Melon seeds were inoculated with ∼10^7^ CFU/ml bacterial suspensions and sown in pots containing sand. The pots were kept in a growth chamber at 28°C and the shoot weight of emerging seedlings was determined after 10 days. Disease severity was determined using a 0 to 7 scale (0, healthy seedlings; 7, dead seedlings) relative to non-inoculated controls, as described in the Section “Materials and Methods.” The strains were the same as for leaf infiltration assays except that the *hrcV* mutant was not included in these assays. Data represent means (15 seedlings per treatment) and standard errors of one representative experiment, out of two with similar results. No significant differences among strains were found by ANOVA and Tukey’s HSD *post hoc* test.

Since pACM6 does not carry evident virulence genes and does not occur in several group I and in all tested group II strains, we hypothesized that this plasmid does not contribute significantly to the virulence of *A. citrulli* M6. To assess this hypothesis, we first carried out inoculation experiments of melon and *Nicotiana benthamiana* leaves using the syringe infiltration method. *N. benthamiana* was included in these assays since it was recently shown that this plant can be used as a surrogate host for studying pathogenicity aspects of *A. citrulli* (Traore et al., 2019). These experiments showed no differences in the progress of symptom appearance and in symptom severity among the tested cured strains, non-cured strains and wild-type M6 (Figure 5A and Supplementary Figure S6). In agreement with these results, no significant differences among the tested strains were observed in melon seed transmission assays (Figure 5B). Overall, these experiments support that, under tested conditions, loss of pACM6 does not affect the virulence ability of *A. citrulli* M6.

## Conclusion

Pacific Biosciences sequencing of the genome of the group I model *A. citrulli* strain M6 allowed its complete assembly and revealed the presence of a ∼53 kb plasmid, which we named pACM6. The occurrence of the plasmid was further confirmed by mapping of contigs of the draft genome of the same strain on the pACM6 sequence, plasmid extraction and electrophoresis, and combination of Southern blot analysis and PCR that demonstrated the circular nature of pACM6. Moreover, we were able to obtain M6-derivative strains in which the plasmid was eliminated. Sequence analyses and PCR tests revealed that pACM6-like plasmids are present in some, but not all group I strains of *A. citrulli*, and likely absent in group II *A. citrulli* strains. The pattern of occurrence of pACM6 and pACM6-like plasmids among group I strains suggests that these plasmids could have been acquired in more than one horizontal transfer event; however, this hypothesis should be further verified. We also showed that pACM6 is present at low average copy numbers in *A. citrulli* M6 cells. While the Fic-VbhA TA module encoded in pACM6 could play a role in maintenance of this plasmid in the bacterial population, further investigation is needed to assess this possibility and to determine the conditions that lead to plasmid loss or changes in plasmid copy number. While experiments reported in this study indicate that pACM6 does not significantly contribute to *A. citrulli* pathogenicity, further studies are needed to determine whether this plasmid contributes to the fitness of the bacterium.

## Materials and Methods

### Bacterial Strains

*Acidovorax citrulli* M6 was isolated in Israel in 2002 from a symptomatic melon fruit (Burdman et al., 2005). Other *A. citrulli* strains used in this study are listed in Table 3. *E. coli* DH5a (Invitrogen, Carlsbad, CA, United States) and *A. citrulli* AAC00-1 (Walcott et al., 2000) were used as negative controls for plasmid visualization. Unless stated otherwise, bacterial strains were grown in nutrient broth (NB, Difco Laboratories, Detroit, MI, United States) with shaking (150 rpm) or on NA (NB containing 15 g/l agar) at 28°C, except for the *E. coli* strain that was grown at 37°C in Luria-Bertani (LB, Difco Laboratories).

### PacBio Genome Sequencing of *A. citrulli* M6

*Acidovorax citrulli* M6 was grown for 24 h in NB medium as described above, and bacterial DNA was isolated with the GenElute^TM^ Bacterial Genomic DNA Kit (Sigma-Aldrich, St. Louis, MO, United States) according to the manufacturer’s instructions. The DNA was sent to Macrogen (Seoul, South Korea) for sequencing. The sample was prepared according to standard instructions for SMRTbell^TM^ templates for sequencing on the PacBio RS System, and sequenced using SMRT^®^ Sequencing. The sequencing yielded a total of 1,363,486,425 polymerase read bases with 101,724 polymerase reads. The read N50 was 19,405 bp, the average read length was 13,403 bp and the polymerase read quality was 0.842 (all values, post-filter). Finally, a total of 192,661 pre-filtered subreads, with an average read length of 7,055 bp, was used for *de novo* assembly.

### Assembly and Annotation of the M6 Genome

For *de novo* assembly, the whole set of sub-reads was used as input for Canu v1.4 (Koren et al., 2017) with the following parameters: -pacbio-raw genomeSize = 4.8 m. The average coverage was assessed by mapping Canu trimmed/corrected reads against the obtained assembly with bwa (Li and Durbin, 2009), samtools (Li et al., 2009), and awk. A previous MiSeq paired-end library (150 bp × 2 and 300 bp insert size; Eckshtain-Levi et al., 2016) was used to correct the Canu consensus using SEQuel v1.0.2 (Ronen et al., 2012) with all parameters set as default except for -v 100. A mate-paired MiSeq library (150 bp × 2 and 2.5 Kb insert size) was mapped against the corrected consensus with bowtie2 (Langmead and Salzberg, 2012) in paired-end mode and the following options: –very-sensitive-local-reorder. Samtools v0.1.19 was used to convert the SAM mapping file to sorted BAM file (Li et al., 2009). The BAM file was imported to Gap5 (Bonfield and Whitwham, 2010), which was used to check for direct repeats longer than 500 bp, mate-paired reads connections and for trimming the obtained consensus. Finally, one of the direct repeats was trimmed to produce both linear consensus of the chromosome and the plasmid. Mummer v3 (nucmer and mummerplot; Kurtz et al., 2004) was used to screen for possible integrative forms of the plasmid. Genome annotation was performed with Prokka v1.12-beta (Seemann, 2014) and the following options: –kingdom Bacteria; –gcode 11; –e-value 1e-9; –rna. The PHAST server was used to annotate prophage sequences (Zhou et al., 2011). InterProScan v5.27-66.0 (Jones et al., 2014) was used to assign GO, TIGR and PFAM terms while PRIAM v2 (-pt 0.8 -mo -1 -1 -mp 70 -cc T -cg T; Claude-Renard et al., 2003) was used to assign enzyme commission numbers (release March 2015) to the *A. citrulli* M6 proteome. OriFinder v1 (Gao and Zhang, 2008) was used to predict the replication origin in the chromosome and in the plasmid. Circos (Krzywinski et al., 2009) was used to plot the chromosome and plasmid features. GC skew and cumulative GC skew were calculated with a custom python script.

### Additional Sequence Analysis Tools

CodonW (Peden, 1999) and tAI R package (dos Reis et al., 2003) were used to compute the Codon Adaptation Index (CAI), the Effective Number of Codons (Nc), and the tRNA Adaptation Index (tAI). Presence of plasmids similar to pACM6 in the genome of *A. citrulli* AAC00-1 and in the draft genome sequences of *A. citrulli* strains from a proprietary collection were performed with the BlastN program implemented on Geneious version 10.1.2 (Biomatters Ltd., Auckland, New Zealand^2^), using the following parameters: maximum E-value 1e-5; gap costs (5, 2, open and extend, respectively); match-mismatch scoring 2–3; maximum hits 200. Then the hits were filtered using the parameters of max sequence length >500 nt, and % pairwise identity >90%. The resulting hits were mapped to pACM6 using the Geneious Mapper. MEGAN (version 6.12.6) was used to assess the phylogenetic assignment of selected plasmid genes following export of BlastP alignments and using standard parameters (Huson et al., 2007). SOS box consensus sequences were identified using the fuzznuc tool^3^ of the EMBOSS package (Rice et al., 2000) using the CTGT-N8-ACAG motif.

### Molecular Biology Techniques

Primers for PCR reactions were designed using Primer3 v.0.4.0 (Koressaar and Remm, 2007; Untergasser et al., 2012), and purchased from Hy Laboratories (Rehovot, Israel). Primers used in PCR and qRT-PCR reactions are described in Supplementary Tables S1, S3, respectively. PCR reactions were performed in an Eppendorf (Hamburg, Germany) Thermal Cycler using REDTaq ready mix (Sigma-Aldrich) in 25-μl reaction volumes, according to manufacturers’ instructions. Unless stated otherwise, PCR products were separated by gel electrophoresis at 120 V for 30 min on a 1% agarose gel in 0.5X Tris-acetate ethylenediaminetetraacetic acid (EDTA) buffer. Gels were stained with 1 μg/ml ethidium bromide and gel images were captured using a C200 gel imaging workstation (Azure Biosystems, Dublin, CA, United States). PCR bands were extracted with the AccuPrep PCR purification kit (Bioneer, Daejeon, South Korea) and sent for sequencing at Hy Laboratories. qRT-PCR reactions were carried out in a LightCycler 480 System (Roche, Basel, Switzerland) with 5xHOT FIREPol^®^ EvaGreen^®^ qPCR Mix Plus (no ROX) (Solis BioDyne, Tartu, Estonia). qPCR reactions were carried out in 10-μl reaction volumes that contained 2 μl of qPCR mix, 0.25 μl of forward primer, 0.25 μl of reverse primer (10 pmol/μl), 2.5 μl of DNA and 5 μl of nuclease-free water. Restriction enzymes were purchased from New England Biolabs (Ipswich, MA, United States).

### Plasmid Confirmation by Southern Blot Analysis and PCR

Total DNA of *A. citrulli* M6 was extracted as described above using the GenElute^TM^ Bacterial Genomic DNA Kit. DNA was digested with restriction enzymes *Sca*I, *Xho*I, and *Hind*III, run by gel electrophoresis as described above for PCR products (except for detection of fragments F1, F2, and F3, for which digestion products were separated in a 0.7% agarose gel for 2 h at 50 V) and transferred to nylon membranes (iBlot DNA Transfer Stack, Invitrogen) for Southern blot hybridizations. Generuler 1-kb DNA Ladder (Fermentas, Burlington, Canada) and aaa*Eco*RI (Thermo Fisher Scientific, Waltham, MA, United States) markers were included. Probes 1 to 4, targeting pACM6 fragments F1 to F5 (Figure 2B,C) were amplified by PCR using primer sets Probe_1 to Probe_4 (Supplementary Table S1). Markers and probes were labeled with a thermostable alkaline phosphatase enzyme (AlkPhos Direct Labeling Reagents, GE Healthcare, Chicago, IL, United States). Southern blot hybridization was carried out using the AlkPhos Direct Labeling Reagents and CDP Start Detection Reagent kits (GE Healthcare) according to manufacturer’s instructions. Bands were visualized using an ImageQuant LAS 500 imaging workstation instrument (GE Healthcare). PCR reactions with primer sets ScaI_F/R, XhoI_F/R, HindIII-1_F/R, HindIII-2_F/R and HindIII-3_F/R (Supplementary Table S1) were carried out to verify the junctions between the different fragments.

### DNA Fingerprinting by Pulse-Field Gel Electrophoresis (PFGE)

Bacterial DNA preparation, digestion with *Spe*I and PFGE were carried out as described (Walcott et al., 2000). Gels were stained for 30 min with 0.5 μg/ml ethidium bromide solution and digital images were captured using an Eagle Eye II Still Video System (Stratagene, La Jolla, CA, United States). Fingerprint profiles for each haplotype were compared using Dice’s (1945) coefficient of analysis using the BioNumerics software package (Applied Math, Kortrijk, Belgium). The unweighted pairwise group method with arithmetic mean (UPGMA) algorithm was used to generate a relatedness dendrogram.

### Quantification of Plasmid Copy Number

Plasmid copy number was determined by qRT-PCR using the methodology described by Wang et al. (2016). *A. citrulli* M6 was grown in NB at 28°C for 24 h and total DNA was extracted with the GenElute^TM^ Bacterial Genomic DNA Kit. Samples were analyzed with primer sets (Supplementary Table S3) specifically targeting three plasmid genes [*ssb* (*APS58_p00013*), *parB1* (*APS58_p00027*), and *vbhA* (*APS58_p00053*)] and three chromosomal genes [*rplN* (*APS58_2372*), *pyrG* (*APS58_3060*), and *smpB* (*APS58_3610*)]. The optimal threshold cycle (Ct) value was determined for all primer pairs by testing different annealing temperatures, and all were set at 61°C. The reaction steps were: 95°C for 15 min; 95°C for 10 s, 61°C for 20 s and 72°C for 20 s (fifty cycles); followed by a melting curve profile from 60 to 95°C, to confirm the specificity of the reactions. Plasmid copy number was defined as the plasmid DNA: chromosome ratio, using the formula 2^-ΔCT^, where ΔCT is the difference in average threshold cycles (Ct) between plasmid and chromosomal genes. Three independent experiments (each one using different cultures and DNA extractions) were carried out.

### Plasmid Curing

*Acidovorax citrulli* M6 was grown for 24 h in tubes containing 3 ml NB, at 28°C with shaking. Bacteria were pelleted by centrifugation (6000 g, 5 min, 4°C), resuspended in 3 ml of fresh NB and incubated for additional 24 h at 28 or 41°C. This step was repeated twice, after which the cultures were serially diluted before plating relevant dilutions onto NA plates. The plates were incubated at 28°C for 2 days, after which colonies were tested by colony-PCR with primer sets targeting the plasmid (Supplementary Table S1) as well as with the *A. citrulli*-specific BX-S primer set targeting a chromosomal region (Bahar et al., 2008). A total of 450 colonies per temperature treatment were tested in three different experiments, and plasmid loss was verified by similar PCR reactions using extracted DNA.

### Virulence Assays

*Acidovorax citrulli* M6 cured strains were tested in leaf infiltration and seed transmission assays. Melon (*Cucumis melo*) cultivar Glory (Origene Seeds, Rehovot, Israel) was used in both assays, while *N. benthamiana* (Goodin et al., 2008) plants were used in leaf infiltration assays. Three cured strains, M6-PC1, M6-PC5, and M6-PC8, which were isolated from three different plasmid curing experiments, were tested in comparison with wild-type M6. In these experiments we also included three isolates, M6-NC1, M6-NC2, and M6-NC3, that were collected at the end of curing experiments after incubation at 41°C, and were shown to retain pACM6. Leaf infiltration assays also included a non-pathogenic M6 mutant defected in the *hrcV* gene and impaired in type III secretion (Bahar and Burdman, 2010). For leaf infiltration, plants were grown in a greenhouse (27–28°C) in 12-cm diameter plastic pots with a peat-based commercial soil (Garden Mix, Rehovot, Israel). Bacteria were grown on NA at 28°C for 24 h and resuspended in 10 mM MgCl_2_ to an optical density at 600 nm (OD_600_) of 0.2 (about 10^8^ CFU/ml) using a Helios Gamma spectrophotometer (Thermo Fisher Scientific). These bacterial suspensions were diluted 100 times with 10 mM MgCl_2_ yielding ∼10^6^ CFU/ml inoculum suspensions. These suspensions were used to inoculate the abaxial parts of the two youngest fully developed leaves of 3- or 5-week-old plants of melon and *N. benthamiana*, respectively, by syringe infiltration. Symptoms were recorded every 24 h. Two independent experiments were carried out with *N. benthamiana* and melon plants. In each experiment, all strains were used to inoculate at least six or eight leaves of *N. benthamiana* and melon, respectively. Seed transmission assays were performed as described by Bahar et al. (2009) with few modifications. Melon seeds (15 seeds per bacterial strain) were incubated for 2 h at room temperature in bacterial suspensions of about 10^7^ CFU/ml that were prepared as described above (but diluting the ∼10^8^ CFU/ml suspensions 10 times). Then, the suspensions were discarded, and the seeds were dried under a laminar flow hood. Seeds were then sown in 11-cm diameter plastic pots containing sand. The plants were grown in a growth chamber (16 h light/8 h dark, 28°C, relative humidity set to 100%). At 10 days after inoculation (d.a.i.), the fresh weight of the plant shoots was determined. Disease severity was scored using a 0 to 7 scale, based on shoot-weight values of inoculated plants relative to the average shoot weight of non-inoculated controls, as defined by Bahar et al. (2009): 0, weight higher than 90% of average control weight; 1 to 5, weight equal to 76–90%, 61–75%, 46–60%, 31–45%, and 16–30% of average control weight, respectively; 6, weight equal to or lower than 15% of average control weight; 7, seedling dead. The data were analyzed using one-way analysis of variance (ANOVA) and the Tukey’s honestly significant difference (HSD) *post hoc* test for mean comparisons using the JMP software (SAS Institute Inc., Cary, NC, United States). Seed transmission assays were carried out twice yielding similar results.

### Additional Methods

Plasmid extraction and visualization, RNA-Seq related methods and growth curve experiments are described in the Supplementary Material.

## Author Contributions

SB, RY, and FPM conceived the research. TR extracted and tested the DNA for sequencing. RY, FPM, IJG, GS, and MZ performed the experiments. DSG did most bioinformatics work. DSG, FPM, RY, IJG, GS, TR, and SB revised the automatic annotation. MZ and GC did the overlap analyses of the plasmid in *A. citrulli* genomes, under the supervision of RW. IJG and IP carried out RNA-Seq analyses. RY, DSG, and SB wrote the draft of the manuscript. All authors edited the manuscript and approved it.

## Conflict of Interest Statement

The authors declare that the research was conducted in the absence of any commercial or financial relationships that could be construed as a potential conflict of interest.

## Footnotes

1 https://www.ncbi.nlm.nih.gov/genome/?term=Acidovorax+citrulli

2 http://www.geneious.com

3 http://www.bioinformatics.nl/cgi-bin/emboss/fuzznuc
