# Supplementary material for: Complete Assembly of the Genome of an Acidovorax citrulli Strain Reveals a Naturally Occurring Plasmid in This Species
Source: Front Microbiol. 2019 Jun 20;10:1400. doi: 10.3389/fmicb.2019.01400 (PMC6595937; doi:10.3389/fmicb.2019.01400)
Supplement: Supplementary file 1 [file Data_Sheet_1.PDF]

Supplementary Material

Complete assembly of the genome of an *Acidovorax citrulli* strain reveals a naturally occurring plasmid in this species

Rongzhi Yang, Diego Santos Garcia, Francisco Pérez Montaño, Gustavo Mateus da Silva, Mei Zhao, Irene Jiménez Guerrero, Tally Rosenberg, Gong Chen, Inbar Plaschkes, Shai Morin, Ron Walcott and Saul Burdman*

*** Correspondence:** Saul Burdman: saul.burdman@mail.huji.ac.il

Supplementary tables

Supplementary Table S1. Primers used to verify the circular nature of *Acidovorax citrulli* M6 pACM6 and to assess the occurrence of pACM6 or pACM6-like plasmids in *A. citrulli* strains.

Primer	5'->3' Sequence	Tm (°C)	Purpose
Probe_1_F	ACTCCACGCACGATCCC	61	Used for the amplification of pACM6 probe 1 (1,039 bp) for detection of plasmid fragments F1 and F2 by Southern blot (see Figure 2)
Probe_1_R	CTCGGTAGCTGAGCTTGG	59	
Probe_2_F	GGATCGTAGATCCTTCGCC	59	Used for the amplification of pACM6 probe 2 (1,147 bp) for detection of plasmid fragment F3 by Southern blot (see Figure 2)
Probe_2_R	ATCCGAGAGCTTGCGAAGG	61	
Probe_3_F	CCGCAGGCCACCTTTCTT	61	Used for the amplification of pACM6 probe 3 (1,060 bp) for detection of plasmid fragment F4 by Southern blot (see Figure 2)
Probe_3_R	AGGCGATTTGCTGCTTCG	60	
Probe_4_F	TCCAGACGCGCATCAACA	61	Used for the amplification of pACM6 probe 4 (1,104 bp) for detection of plasmid fragment F5 by Southern blot (see Figure 2)
Probe_4_R	TATTTGCGCATGCTCGCCT	61	

ScaI_F	TCAAGCTGCGTGAGAACGG	62	Used for the amplification of a 629-bp product spanning the *Sca*I site between pACM6 fragments F1 and F5 (see Figure 2)
ScaI_R	CACACCGTTCTTGTCGCC	61	
XhoI_F	AAGGCACCTGGCAGCTGC	66	Used for the amplification of a 561-bp product spanning the *Xho*I site between pACM6 fragments F1 and F2 (see Figure 2)
XhoI_R	ACCGCCATGTCGCTAGCC	65	
HindIII-1_F	TTATTGGCGGCACAGGGC	63	Used for the amplification of a 631-bp product spanning the *Hind*III (1) site between pACM6 fragments F2 and F3 (see Figure 2)
HindIII-1_R	GACGGACTTGGTGGCCTTG	63	
HindIII-2_F	GTCCTCATGCCCTCTCCG	62	Used for the amplification of a 566-bp product spanning the *Hind*III (2) site between pACM6 fragments F3 and F4 (see Figure 2)
HindIII-2_R	GAGTTCAATGCACTGCATCC	60	
HindIII-3_F	AGACAAGCCACCACGCAAA	62	Used for the amplification of a 491-bp product spanning the *Hind*III (3) site between pACM6 fragments F4 and F5 (see Figure 2)
HindIII-3_R	TTGCCATCGCCCTCAATGT	61	
pACM6-F1	GTAGCGGTCGGAATCTTCTT	57	Used for the amplification of a 585-bp product to verify presence of pACM6 or like-plasmids in *A. citrulli* strains
pACM6-R1	AGTACAGCTCGTTCTTGAGC	58	
pACM6-F2	AACCATGAAGGACCTCCAGA	58	Used for the amplification of a 671-bp product to verify presence of pACM6 or like-plasmids in *A. citrulli* strains
pACM6-R2	CGTACCCCGTGAAGAAAGT	57	
BX-L1	CAGCTGGGAGCGATCTTCAT	63	BX-S primer set; specific primers used for diagnostics of *A. citrulli*, amplifying a 279-bp chromosomal product (Bahar et al., 2008)
BX-S-R2	GCGTCAGGAGGGTGAGTAGCA	65	

Supplementary Table S2. Assignment of nine plasmid proteins of pACM6 following MEGAN analysis.

**Protein ID**	**Product**	**Assignment**	**Top hits** [1]
APS58_p00051	ParA	Order: Burholderiales	*Burkholderia cenocepacia*, *Paraburkholderia ferrarieae*, *Advenella* sp., *Curvibacter gracillis*, Burkholderiales, *Acidovorax* sp., *Lautropia* sp.
APS58_p00025	StbB	Class: Betaproteobacteria	*Pelomonas* sp., *Methylibium* sp., *Rhizobacter* sp., *Hydrogenophaga* sp., *Ideonella* sp., *Roseateles depolymerans*, Burkholderiales bacterium, *Piscinibacter* sp., *Acidovorax temperans*
APS58_p00056	UmuD	Class: Betaproteobacteria	*Polaromonas jejuensis*, *Ottowia thiooxydans*, Betaproteobacteria bacterium, *Hydrogenophaga* sp., *Aquabacterium parvum*, *Aquabacterium olei*, *Hydrogenophaga intermedia*
APS58_p00057	DinB	Class: Betaproteobacteria	*Pseudorhodoferax* sp., *Acidovorax* sp., *Variovorax paradoxus*, *Acidovorax radicis*, *Acidovorax temperans*, *Acidovorax carolinensis*, *Acidovorax wautersii*, *Ottowia thiooxydans*, *Curvibacter lanceolatus*, *Diaphorobacter polyhydroxybutyrativorans*
APS58_p00013	Ssb	Phylum: Proteobacteria	*Acidovorax avenae*, *Xanthomonas citri*, *X. axonopodis* pv. *cajani*, *Acidovorax valerianellae*, *Xanthomonas citri* pv. *citri*, *Xanthomonas oryzae* pv. *oryzicola*, *Pseudomonas* sp.
APS58_p00023	MobC	Phylum: Proteobacteria	*Burkholderia vietnamiensis*, *Ralstonia pickettii*, *Asticcacaulis benevestitus*, Burkholderiales, *Pseudomonas panipatensis*, *Azoarcus* sp., *Bordetella parapertussis*, *Xanthomonas albilineans*
APS58_p00027	ParB_1	Phylum: Proteobacteria	*Nitrosospira multiformis*, *Alcaligenes faecalis*, *Acidovorax soli*, *Burkholderia cenocepacia*, *Pseudomonas aeruginosa*, *Nitrosospira lacus*, *Ralstonia solanacearum*, *Hydrogenophaga* sp., *Rhizobacter* sp., *Xenophilus azovorans*
APS58_p00047	RepA	Phylum: Proteobacteria	*Legionella* sp., *Acidovorax* sp., Nitrosomonadaceae, *Pseudomonas aeruginosa*, *Nitrosopira multiformis*, *Burkholderia diffusa*, *Nitrosovibrio* sp., *Acidovorax* sp., *Rhodanobacter* sp., *Caballeronia sordidicola*, *Alicycliphilus* sp.
APS58_p00050	ParB_2	Phylum: Proteobacteria	*Variovorax paradoxus*, *Nitrosomonas eutropha*, *Thioalkalivibrio* sp., *Alicycliphilus* sp., *Polynucleobacter* sp., *Methylobacter whittenburyi*, *Massilia putida*, *Thioalkalivibrio* sp.

[1] Black text: Class Betaproteobacteria, Order Burkholderiales. Gray text: Class Betaproteobacteria, Order Nitrosomonadales or Rhodocyclales. Red text: Class Gammaproteobacteria. Green text: Class Alphaproteobacteria.

Supplementary Table S3. Primers used in quantitative (real-time) PCR (qRT-PCR) for assessment of pACM6 copy number.

Primer	5'->3' Sequence	Purpose
ssb_F	GGCTTCTGGTTACCTGTGGA	Used for amplification of a 180 bp product within the plasmid *ssb* (*APS58_p00013*) ORF
ssb_R	GTAAGGCAGGATGGCAACAC	
parB1_F	GATACGACGGTGCAAAGGAT	Used for amplification of a 181 bp product within the plasmid *parB1* (*APS58_p00027*) ORF
parB1_R	GTTTGCTCCAGAACCTGCAT	
vbhA_F	GCGCTGAGCCTTAGAGAAGA	Used for amplification of a 174 bp product within the plasmid *vbhA* (*APS58_p00053*) ORF
vbhA_R	CTCCATACCAGCTGCCGTA	
rplN_F	GAGGTTTACAGCGCAGTGGT	Used for amplification of a 189 bp product within the chromosomal *rplN* (*APS58_2372*) ORF
rplN_R	AGCCAGGGACACGATCTTC	
pyrG_F	GTGCAGATCTTGCCTTCCAC	Used for amplification of a 189 bp product within the chromosomal *pyrG* (*APS58_3060*) ORF
pyrG_R	CGACAGCTACAAGTCGGTGA	
smpB_F	TCTTCAGGGGATTGATCTGG	Used for the amplification of a 185 bp product within the chromosomal *smpb* (*APS58_3610*) ORF
smpB_R	CATCGCCGACAACAAGAAG	

Supplementary figures

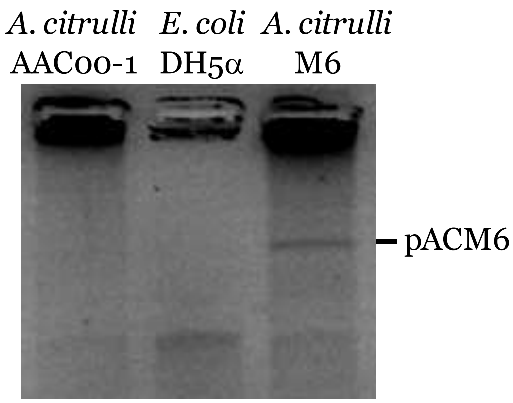

Supplementary Figure S1. Detection of the *Acidovorax citrulli* pACM6 plasmid. Plasmid extraction and visualization were carried out using the Birnboim and Doly (1979) procedure as described in Materials and Methods. Samples: *A. citrulli* AAC00-1, *Escherichia coli* DH5α (negative controls) and *A. citrulli* M6. The lower bands observed in all samples correspond to residual RNA and DNA debris.

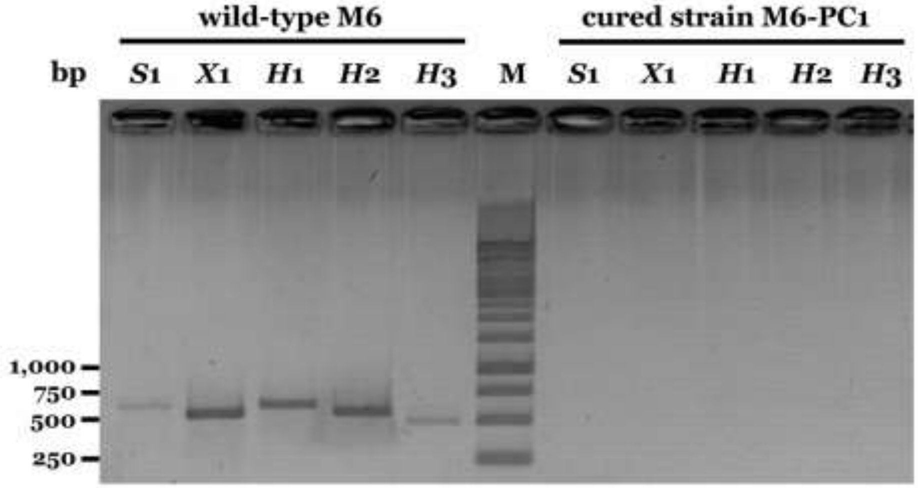

Supplementary Figure S2. Verification of junction regions between pACM6 fragments resulting from restriction of *A. citrulli* M6 DNA with enzymes *Sca*I, *Xho*I and *Hind*III. Genomic DNA of *A. citrulli* M6 (left) and an M6-cured strain, M6-PC1 (right), were used as template for PCR amplification with primers ScaI_F/ScaI_R (*S*1), XhoI_F/XhoI_R (*X*1), HindIII-1_F/HindIII-1_R (*H*1), HindIII-2_F/HindIII-2_R (*H*2) and HindIII-3_F/HindIII-3_R (*H*3). PCR targets and primer details are shown in Figure 2B and Supplementary Table S1, respectively. The expected length of the PCR products are: *S*1, 629 bp; *X*1, 561 bp; *H*1, 631 bp; *H*2, 566 bp; and *H*3, 491 bp. M, Generuler 1-kb DNA Ladder (Fermentas). The PCR products obtained from M6 DNA were verified by sequencing and matched with the expected sequences in pACM6.

Strain 213-58 (B5; 92.8%)

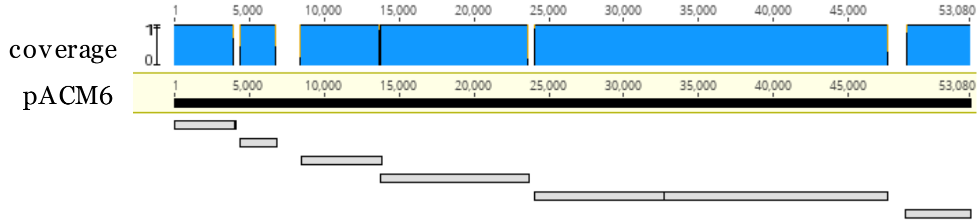

Strain 213-61 (B5; 92.1%)

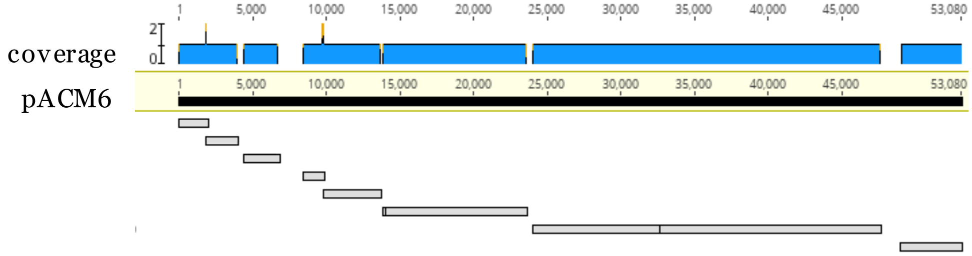

Strain 213-55 (B9; 92.5%)

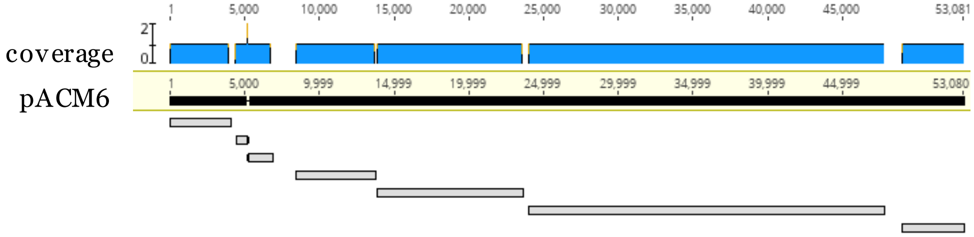

Strain 213-54 (B12; 83.8%)

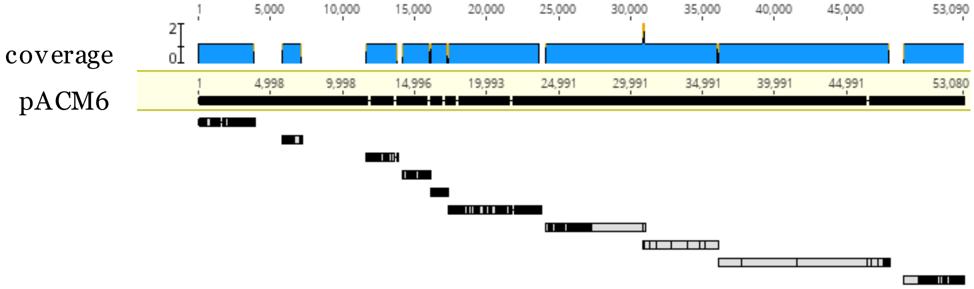

Supplementary Figure S3. Overlap of the *Acidovorax citrulli* pACM6 plasmid with draft genomes of representative group I strains. The pACM6 sequence was used as query in BlastN analyses (Geneious 10.1.2) against strain contigs as described in Materials and Methods. The pulse field gel electrophoresis (PFGE) haplotype and the percentage of overlap are indicated for each strain. The coverage of each position in pACM6 by the contigs is indicated above the plasmid sequence. The strain contigs are represented by gray bars below the pACM6 sequence. Black bars/regions in the contig bars indicate presence of single nucleotide polymorphism (SNP).

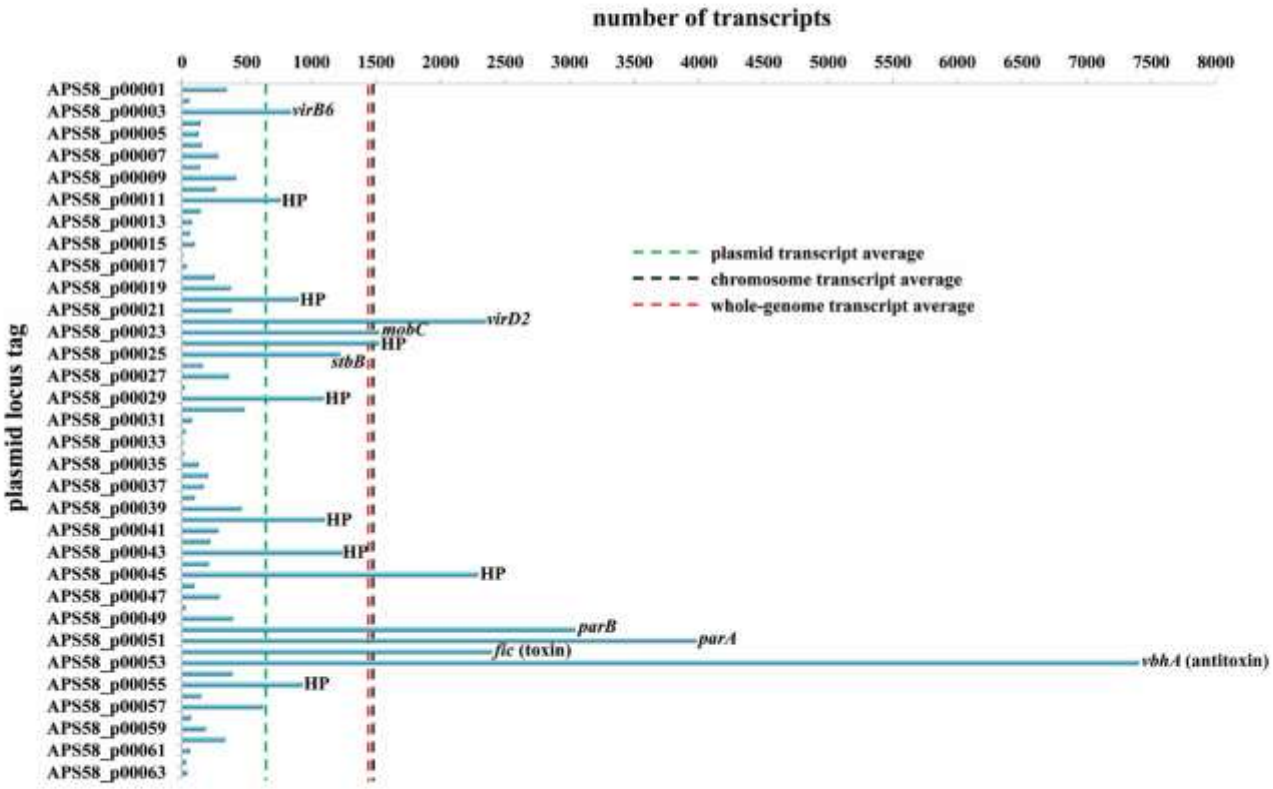

Supplementary Figure S4. **Gene expression of pACM6 genes.** The plot shows the number of transcripts obtained for each open reading frame (ORF) of pACM6 following RNA-Seq of *A. citrulli* M6 after 72 h of growth in XVM2 minimal medium at 28°C. Data are normalized means of three independent samples. Vertical dashed lines indicate the average number of transcripts per ORF of pACM6 (green), chromosome (black), and whole genome (red). Gene names are shown for genes whose number of transcripts was higher than the average number of transcripts per ORF of pACM6. HP, hypothetical protein.

A

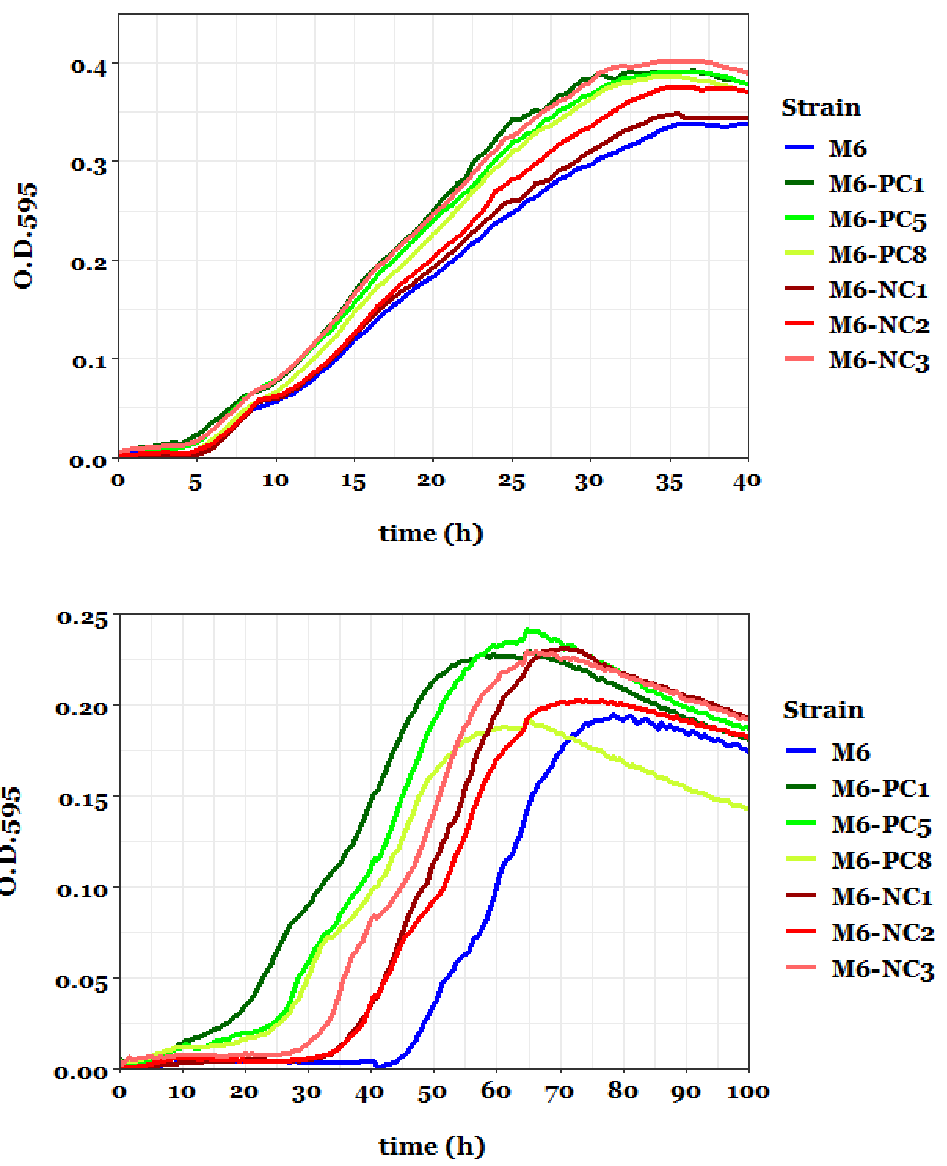

Supplementary Figure S5. Growth curves of *A. citrulli* strains in rich and minimal media. Wild-type M6 and cured strains M6-PC1, M6-PC5 and M6-PC8 were grown in rich (nutrient broth, NB; **A**) and minimal (XVM2; **B**) media at 28°C in a plate reader. Optimal density (595 ηm) values were measured every 15 min. Isolates M6-NC1, M6-NC2 and M6-NC3, that were collected from curing experiments after exposure to 41°C and retained the plasmid, were also included. Data are shown for one experiment with three replicates per strain, out of two experiments with similar results.

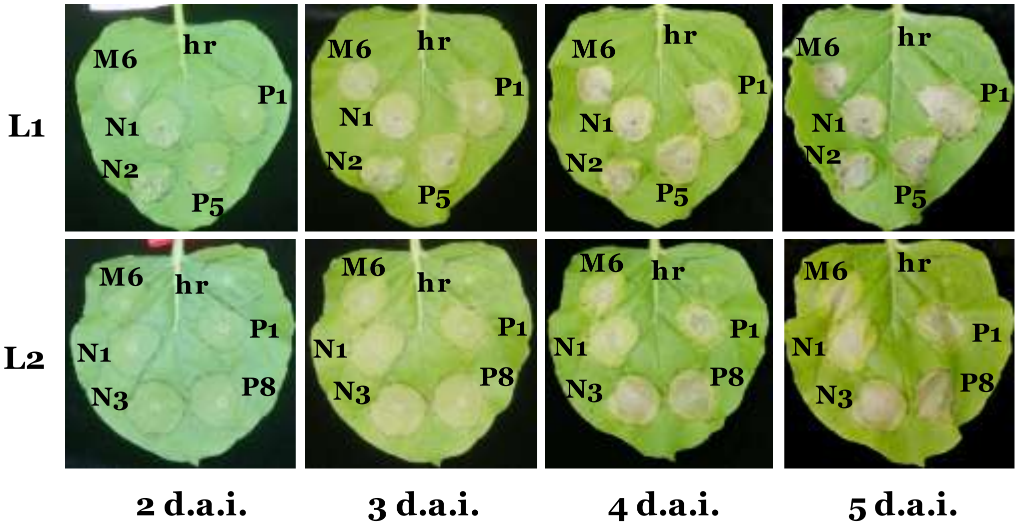

Supplementary Figure S6. *Nicotiana benthamiana* leaves inoculated with *A. citrulli* strains. Leaves of five-week-old *N. benthamiana* plants were syringe-infiltrated in their abaxial side with 10^6 CFU/ml suspensions of *A. citrulli* M6 (M6), M6 cured strains M6-PC1 (P1), M6-PC5 (P5) and M6-PC8 (P8), non-cured strains (that were exposed to the 41°C treatment and retained pACM6) M6-NC1 (N1), M6-NC2 (N2) and M6-NC3 (N3), and an M6 mutant defected in the *hrcV* gene and impaired in pathogenicity (hr). The plants were kept in a greenhouse at 27-28°C. Symptoms were recorded every day [since 2 days after inoculation (d.a.i.) when symptoms became visible] until 5 d.a.i. Pictures are shown for two representative leaves (L1 and L2) of one experiment out of two experiments with similar results. In each experiment, each of the cured and non-cured strains were used for inoculation of at least 6 leaves, while wild-type M6 and the *hrcV* mutant were used for inoculation of all leaves.

Detailed information of additional methods

Plasmid extraction and visualization. Bacteria were grown overnight in 5 ml NB or LB media (for *A. citrulli* and *E. coli*, respectively). Cells were pelleted by centrifugation (6000 g, 5 min, 4°C) and plasmid DNA was extracted following selective alkaline denaturation of chromosomal DNA using the method described by Birnboim and Doly (1979). Plasmid DNA samples were mixed with 5X sample buffer [25% sucrose, 5 mM sodium acetate, 0.05% bromophenol blue, 0.1% SDS] and run in a 0.8% agarose gel for 3 h at 100 V and 400 mA, with an electrophoresis buffer that contained 40 mM Tris (Trizma Base), 20 mM sodium acetate and 2 mM EDTA (adjusted to pH 7.8 with acetic acid). Gels were stained with ethidium bromide and visualized as described above.

RNA isolation, RNA sequencing and mapping. *Acidovorax citrulli* M6 was grown in 5 ml XVM2 minimal medium (Wengelnik et al. 1996) at 28ºC for 72 h. Total RNA was isolated using TRI Reagent (Sigma-Aldrich) and Direct-zol RNA miniprep kit (Zymo Research, Irvine, CA, USA). Samples were treated with RNase free DNase using the Turbo DNA-free kit (Invitrogen), and RNA concentration was determined using Nanodrop DS-11 FX (DeNovix, Wilmington, DE, USA, USA). Ribosomal RNA was depleted using the MICROBExpress Bacterial mRNA Enrichment kit (Thermo Fisher Scientific). The integrity and quality of the ribosomal depleted RNA was checked by gel electrophoresis in a 1% agarose gel and with Agilent Bioanalyzer 2100 (Agilent Technologies, Santa Clara, CA, USA). Three independent RNA extractions were obtained. RNA-Seq was carried out by

the Center for Genomic Technologies (The Hebrew University of Jerusalem) with a NextSeq 2000 sequencing instrument (Illumina, San Diego, CA, USA). Ribosomal RNA-depleted samples were used to generate whole transcriptome libraries with the NextSeq 500 high output kit (Illumina) following manufacturer recommendations. The quality of the amplified cDNA was analyzed using an Agilent 2200 TapeStation system and quantified using a Qubit 2.0 fluorimeter (Invitrogen). Raw reads were quality checked with FastQC v0.11.4 and trimmed with Cutadapt v1.12 using default parameters (Martin, 2011). The reads were then mapped to the *A. citrulli* M6 genome using STAR v2.201 (Dobin et al., 2013). Approximately 10 million reads per sample were mapped and mapping files were further processed for visualization by samtools v0.1.19. The resulting Bam files were used to improve gene predictions along the genome using cufflinks v2.2.1 followed by cuffmerge without any guiding reference file (Trapnell et al., 2010; Roberts et al., 2011). The number of transcripts per gene were normalized with DESeq2 v3.7 using the median-of-ratio normalization method (Love et al., 2014) using R (R Development Core Team, 2011).

Growth curve experiments. *Acidovorax citrulli* strains were grown overnight in NB medium as described in Materials and Methods. Bacteria were pelleted by centrifugation (6000 g, 5 min, 4°C) and resuspended in fresh NB or XVM2 to an OD_{600} of 0.2. These suspensions were 1:100-diluted with NB and XVM2, respectively, and 1 ml-samples were loaded into the wells of a Nunclon Δ Surface 24-well polystyrene multidish (Nunc, Roskilde, Denmark). The plates were incubated in an Infinite F200 plater reader (Tecan, Männedorf, Zwitzerland) at 28°C, with 10-s shaking every 30 min. Bacterial growth (OD_{595}) was measured every 15 min for 40 and 100 h, for NB and XVM2 cultures, respectively. This experiment was carried out twice for each medium, each time with 3 replicates per strain.

References

Birnboim, H., and Doly, J. (1979). A rapid alkaline extraction procedure for screening recombinant plasmid DNA. *Nucleic Acids Res.* 7, 1513-1523.

Dobin, A., Davis, C. A., Schlesinger, F., Drenkow, J., Zaleski, C., Jha, S., et al. (2013). STAR: ultrafast universal RNA-seq aligner. *Bioinformatics* 29, 15-21.

Love, M.I., Huber, W., and Anders, S. (2014). Moderated estimation of fold change and dispersion for RNA-Seq data with DESeq2. *Genome Biol.* 15, 550. doi: https://doi.org/10.1186/s13059-014-0550-8

Martin, M. (2011). Cutadapt removes adapter sequences from high-throughput sequencing reads. *EMBnet J.* 17, 10-12. doi:https://doi.org/10.14806/ej.17.1.200

R Development Core Team. (2011). R: A language and environment for statistical computing. Viennam Austria: The R Foundation for Statistical Computing.

Roberts, A., Pimentel, H., Trapnell, C., and Pachter, L. (2011). Identification of novel transcripts in annotated genomes using RNA-Seq. *Bioinformatics* 27. 2325-2329.

Trapnell, C., Williams, B., Pertea, G., Mortazavi, A., Kwan, G., van Baren, J., et al. (2010). Transcript assembly and quantification by RNA-Seq reveals unannotated transcripts and isoform switching during cell differentiation. *Nat. Biotechnol.* 28, 511-515.

Wengelnik, K., Marie, C., Russel, M., and Bonas, U. (1996) Expression and localization of HrpA1, a protein of *Xanthomonas campestris* pv. *vesicatoria* essential for pathogenicity and induction of the hypersensitive reaction. *J. Bacteriol.* 178, 1061–1069.
